# Full-Length Computational Model of the SARS-CoV-2 Spike Protein and Its Implications for a Viral Membrane Fusion Mechanism

**DOI:** 10.3390/v13061126

**Published:** 2021-06-11

**Authors:** Wataru Nishima, Marta Kulik

**Affiliations:** 1 New Mexico Consortium, Los Alamos, NM 87545, USA; 2 University of New Mexico, Albuquerque, NM 87131, USA; 3 Biological and Chemical Research Centre, Department of Chemistry, University of Warsaw, Żwirki i Wigury 101, 02-839 Warsaw, Poland; m.kulik@uw.edu.pl

**Keywords:** SARS-CoV-2, coronavirus, spike protein, S protein, membrane fusion, fusion mechanism, viral entry, COVID-19

## Abstract

The SARS-CoV-2 virus has now become one of the greatest causes of infectious death and morbidity since the 1918 flu pandemic. Substantial and unprecedented progress has been made in the elucidation of the viral infection process in a short time; however, our understanding of the structure–function dynamics of the spike protein during the membrane fusion process and viral uptake remains incomplete. Employing computational approaches, we use full-length structural models of the SARS-CoV-2 spike protein integrating Cryo-EM images and biophysical properties, which fill the gaps in our understanding. We propose a membrane fusion model incorporating structural transitions associated with the proteolytic processing of the spike protein, which initiates and regulates a series of events to facilitate membrane fusion and viral genome uptake. The membrane fusion mechanism highlights the notable role of the S1 subunit and eventual mature spike protein uptake through the host membrane. Our comprehensive view accounts for distinct neutralizing antibody binding effects targeting the spike protein and the enhanced infectivity of the SARS-CoV-2 variant.

## 1. Introduction

Severe acute respiratory syndrome coronavirus 2 (SARS-CoV-2) emerged in December 2019 and has rapidly spread throughout the world due to its high rates of transmission and infectivity. As of May 2021, the COVID-19 outbreak has resulted in over 152 million cases and 3.2 million deaths worldwide. The situation globally has implications for both public health and the economy. Although several effective vaccines and antiviral drugs against COVID-19 have been approved, additional measures for COVID-19 prevention and treatment are desired.

Coronaviruses (CoVs) are enveloped, positive-sense, unsegmented, single-stranded RNA viruses belonging to the *Betacoronavirus* genus, including the well-known severe acute respiratory syndrome CoVs (SARS-CoV-1) and Middle East respiratory syndrome CoVs (MERS-CoV), which have approximately 79% and 50% sequence identity with SARS-CoV-2, respectively [1]. Phylogenetic analysis of the whole genome shows that SARS-CoV-2 is 96% identical to the bat coronavirus, which is proposed to be the origin of human SARS-CoV-2 [1].

The viral infection process is initiated when the viral spike protein binds to its hosts’ cognate receptor(s) and induces membrane fusion to deliver the viral RNA into the host cell. The spike protein is encoded in the second ORF and comprises 1159–1363 residues in the *Betacoronavirus* genus [2,3]. Two proteolytic cleavage sites, S1/S2 and S2′, divide the spike protein into three subunits: S1, S1/S2-S2′ and S2′. The cleavage events are thought to trigger a large structural transition from the pre-fusion state to post-fusion state, which involves membrane fusion, merging the viral and host membranes. The overall coronavirus spike proteins are highly conserved among the genus [3]. For example, the SARS-CoV-2 spike protein exhibits 76% sequence identity to the SARS-CoV-1 spike protein. However, there are critical structural differences between coronavirus spike proteins that presumably may confer differences in infectivity. These structural differences include the location of the receptor binding domain (RBD) and S1/S2 cleavage site. For example, RBDs of SARS-CoV-1 and MERS-CoV/HKU4 are situated at the N-terminal domain of S1, while those of MHV and BCoV/OC43 are located at the C-terminal domain of S1. Significantly, the SARS-CoV-1 spike protein recognizes the human ACE2 receptor, while the MERS-CoV/HKU4, MHV, and BCoV/OC43 spike proteins bind to the DPP4 and CEACAM1 receptors and to glycans, respectively [4].

In addition to earlier coronavirus studies, a massive number of SARS-CoV-2 studies have been undertaken in various fields in a short time. Notably, several effective vaccines and therapeutics against COVID-19 have already been approved and are being distributed through unprecedented cooperative efforts. One of the successful LNP-mRNA-based vaccines, BNT162b2, developed by BioNtech/Pfizer, uses a modified full-length spike protein with two proline substitutions (K986P and V987P) [5]. These substitutions inhibit the structural transition of the spike protein from the pre-fusion state to the post-fusion state and maintain the pre-fusion (inactive) state. The ability of proline substitutions to stabilize spike proteins is inherited from HIV-1, MERS-CoV, RSV, and SARS-CoV-1; thus, a greater understanding of the structural and functional transition of the SARS-CoV-2 spike protein would have biomedical relevance for the design of next-generation vaccines and therapeutics against COVID-19. To date, however, the knowledge of SARS-CoV-2 is still fragmented, despite the massive efforts being made and numerous studies being performed [6], and there is a lack of a comprehensive understanding of the viral infection mechanism.

In this study, we report the full-length computational models for the SARS-CoV-2 spike protein in pre- and post-transition states, taking into account the Cryo-EM images and biophysical and fusogenic properties of spike protein segments. The obtained structures provide the mechanistic constraints of multiple structural transitions and further functional insights. Based on these structural models, we propose a viral membrane fusion model for SARS-CoV-2 that consistently accounts for the fragmented knowledge of SARS-CoV-2 and provides a comprehensive view of the viral infection mechanism.

## 2. Results and Discussion

### 2.1. Assignment of Spike Protein Segments is Functionally Crucial for the Viral Membrane Fusion

**Figure 1:**
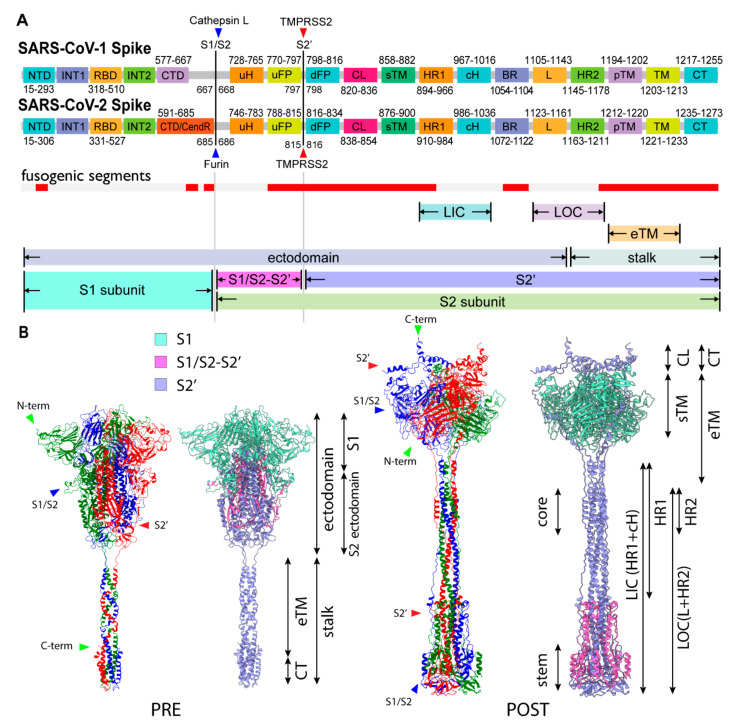
Functional fragments and structural overview of spike protein for SARS-CoV-1/2. (**A**) Schematic segments of S1 and S2 subunits and corresponding residues for SARS-CoV-1/2 (NTD: N-terminal domain; RBD: receptor binding domain; INT1/2: internal sub domain1/2; CTD: C-terminal domain; CendR: multibasic motif; uH: upstream helix; uFP: upstream fusion peptide; dFP: downstream fusion peptide (aka. FP + FPPR); CL: cytoplasmic loop; sTM: sub-transmembrane; HR1: heptad repeat 1; cH: central helix; BR: *β*-rich region; L: linker; HR2: heptad repeat 2; pTM: pre-transmembrane; TM: transmembrane; CT: cytoplasmic tail). Fusogenic segments are shown in the red bar (Table S2 for details), as well as larger regions of multiple segments (LIC: long inner core = HR1 + cH; LOC: long outer core = L + HR2; eTM: extended transmembrane = pTM + TM). (**B**) Structural overview of PRE and POST states (left: RGB colors indicate each monomer; right: light cyan, light magenta, light blue indicate S1, S1/S2-S2′, S2′ subunits, respectively.)

The viral membrane fusion process involves a complex structural transition of the spike protein and proteolytic cleavages at the spike protein S1/S2 and S2′ sites. Each domain of the spike protein plays a distinctive role during the process. Although the region is typically divided according to the structural and functional characteristics, there is no clear consensus on the assignment. This could in part be attributed to the major structural reorganizations of these segments that occur during spike protein maturation and conformational changes. To address these challenges, we have identified segments that participate in distinctive states by considering the biophysical, functional, and structural properties, as well as a conventional naming scheme. These segments include members of the transmembrane region (TM: transmembrane; pTM: pre-transmembrane; sTM: sub-transmembrane), fusion peptide region (uFP: upstream fusion peptide; dFP: downstream fusion peptide), and cytoplasmic region (CL: cytoplasmic loop; CT: cytoplasmic tail). The membrane fusion involves a major structural transition, and accordingly its membrane protein environment is also expected to change significantly. To understand the fusogenic activity of the spike protein to the membrane, we curated associated segments from the literature (Table S2) and also used transmembrane prediction programs to further delineate regions for the S2′ subunit (Figure S1). We identified a probable transmembrane region spanning from residue 1212 to residue 1233 in the C-term region of S2′. This region was further divided into a solid TM region and adjacent aromatic-rich pre-transmembrane region (pTM). An additional putative internal TM region (sTM) was also identified in the S2′ subunit (Figure S1). The widely accepted dFP (residues 816–834) at the N-term of S2′ subunit, a part of a longer FP, is divided by the cleavage at the S2′ site (dFP, uFP). The C-term of S2′ is known as the cytoplasmic tail (CT). Additionally, we defined a relatively hydrophilic region located in between dFP and sTM as a cytoplasmic loop (CL). As described later, its unique profile of hydrophilicity changes provides the structural flexibility and significant function of the CL. Mutagenesis analyses in previous studies have indicated that the uFP, dFP, pTM, CT, and CL segments all have fusogenic activities (Table S2). In summary, the spike protein is divided into the transmembrane (hydrophobic), fusion peptide (amphiphilic), and cytoplasmic (hydrophilic) regions in terms of hydrophobicity. The assignment of the functional segments for SARS-CoV-1/2 is shown in Figure 1A.

### 2.2. Spike Transmembrane in the PRE State Is Characterized by Left-Handed Coiled-Coil Winding

The structures of SARS-CoV-2 spike protein are traditionally classified into the pre-and post-fusion states (PRE and POST states, respectively). This is attributed to the fact that only those two states of structures are resolved in class I viral fusion proteins, and those states are traditionally thought to occur before and after viral membrane fusion, respectively. Unless otherwise noted, we use the term ‘structural transition’ to indicate the major structural change from the compact structure of the spike protein (PRE) to the extended structure (POST-like). In the PRE state, cH and HR1 are compactly folded and each of the RBDs in S1 subunit is able to fluctuate between ‘Up’ and ‘Down’ forms independently. The majority of human cell surface receptors and a number of antibodies are known to bind to the RBD Up form. In addition to the short loop regions, including the S1/S2 cleavage sites and CL, a large portion of the stalk (HR2, pTM, TM, CT) is widely disordered. Although no atomic resolution of the SARS-CoV-2 stalk structure including transmembrane (TM) is available, Cryo-ET studies have revealed that the stalk trimer is highly flexible and asymmetrically bends at three hinges (hip, knee, ankle) [7], while the ectodomain is also frequently tilted relative to the normal axis of the viral envelope [8].

**Figure 2:**
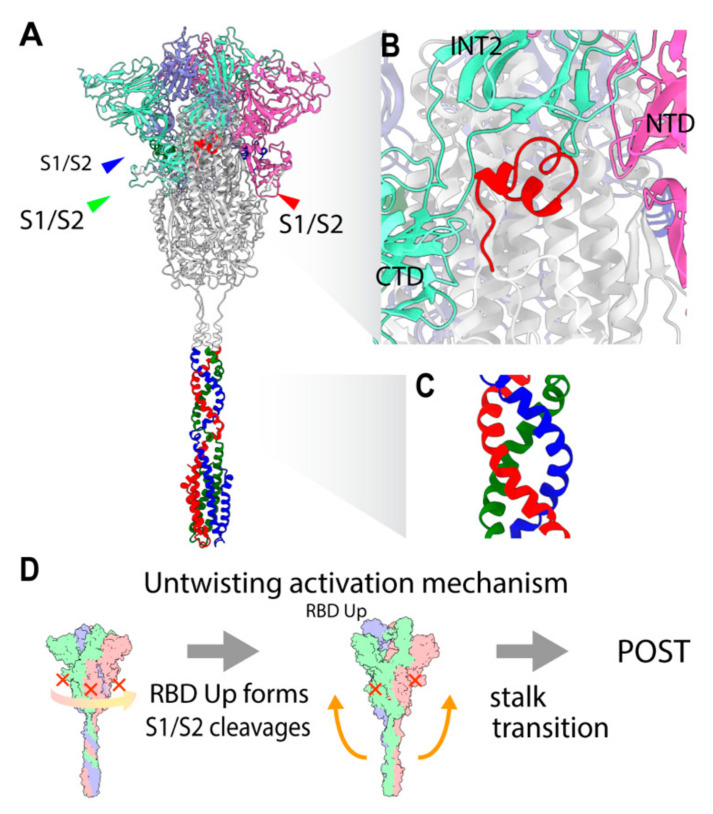
Structural characteristics of the PRE state and untwisting activation mechanism. (**A**) Overview of PRE state structure. The S1 subunits and stalk are colored with light RGB and RGB, respectively. A CL segment is colored in red. (**B**) A close-up of the CL switch (red). CL is making contact with adjacent S1 (light green) while having space with an intra-monomer NTD (light red). CL is disordered in many PRE structures. (**C**) A close-up of the stalk. (**D**) Schematic figure of the untwisting activation mechanism for the transition toward the POST state. The conditions for the transition are three S1/S2 cleavages and three RBD Up forms.

To model a full-length spike structure in the PRE state, we assembled a computational model of the S2′ stalk and Cryo-EM atomic model (PDB: 6XR8) of S1, S1/S2-S2′, and S2′ N-term subunits (Figure 1B and Figure 2A) using a hybridization modeling approach with a C3 trimeric constraint (Materials and Methods). The obtained spike stalk model exhibited three hinges and a partially winded coiled-coil structure oriented counter-clockwise from the N- to the C-term direction (Figure 2A,C). The left-handed coiled-coil in the PRE state stalk is a common feature among the reported computational models (Table S3), which is also consistent with the solution nuclear magnetic resonance (NMR) structure (PDB: 2FXP) of SARS-CoV-1 [9].

### 2.3. Three S1/S2 Cleavages and Three Up RBDs Enable S2 Ectodomain to Rotate for the Structural Transition

Interestingly, CL is disordered in many Cryo-EM PRE structures, except for the ‘locked’ conformation, where it is notably stabilized with three RBD Down forms [10,11]. In the resolved structures of the ‘locked’ conformation, the 833–855 motif in the CL blocks the RBD transition to the Up form (PDB: 6XR8, 6ZOZ, 6ZP2, 6ZGI, 7DF3). The two distinctive states of ‘locked’ and ‘unlocked’ states revealed that the RBD forms also correlate the S1 subunit attachment to S2. The interface between S1 and S2 is closer and the S1 trimer is strongly bound to S2 in the Down form, while the S1 trimer is weakly attached to S2 in the Up form [10,12]. The flexible and protruded CL has been proposed to act as a switch to regulate the RBD Up and Down forms [13], also depending on pH [11]. The Up form implicates the opening of S1, which is an extension of the relative position between NTD and RBD, resulting in a reduction of contacts at the S1–S2 interface [14]. Importantly, the protruding region in the CL blocks the counter-clockwise rotation of S1 around a 3-fold axis by a steric hindrance with an intermonomer CTD in S1, while it has more space with an intramonomer NTD in S1 (Figure 2B). The 3-fold symmetrical S1 trimer seems to be stable when leaving the trimeric interacting ring of S1s [14]. In a structural study of MERS-CoV, the S1s with Up forms were reported to dissociate from S2 as a trimer in the absence of ACE2, suggesting that the S1 trimer association interaction is stronger than that of the S1–S2 interface [15]. This also implies that S1 dissociates from S2 as a trimer [16].

Intriguingly, several studies have reported the rotation of the S1 trimer around the 3-fold axis depending on the degree of the RBD Up or Down forms in the PRE state in SARS-CoV-1/2, while the RDB motion from Down to Up appears to correlate with the counter-clockwise rotation (Table S4). Obviously, when the S1/S2s are uncleaved, the S1/S2 bonds are also one of the significant obstacles for S1 trimers rotating largely in either direction. Comparing between S1/S2 uncleaved and cleaved structures, the S1 trimer rotates clockwise slightly when S1/S2 is cleaved, resulting in the release of CL hindrance against S1 [10].

There are two important structural constraints for the structural transition toward the POST state. First, from the TM fixed frame, the S2 ectodomain has to rotate clockwise from the host to unwind the coiled-coil twist in the stalk to allow disassembly into monomers for the large movement. The rotation of the S2 ectodomain is still structurally feasible while both the TM and S1 trimer are relatively fixed and interacting with the viral and host membranes. Second, the peptide bonds between S1 and S2 subunits constrain the rotation of the S2 ectodomain. Only after the three sites in the trimers are cleaved are the relative rotation between the S1 trimer and S2 and the extension of the spike protein possible. Thus, only with the three S1/S2 cleavages and three RBD Up forms can the S2 ectodomain rotate clockwise, unwinding the stalk. The large movement of the stalk is structurally inhibited by the winding. We hypothesize that the large movement of the stalk is triggered by untwisting of the stalk (untwisting activation mechanism), which requires three S1/S2 cleavages and three RBD Up forms as the condition (Figure 2D).

### 2.4. S2 is Capable of Maintaining the Attachment with an S1 Trimer in Solution

**Figure 3:**
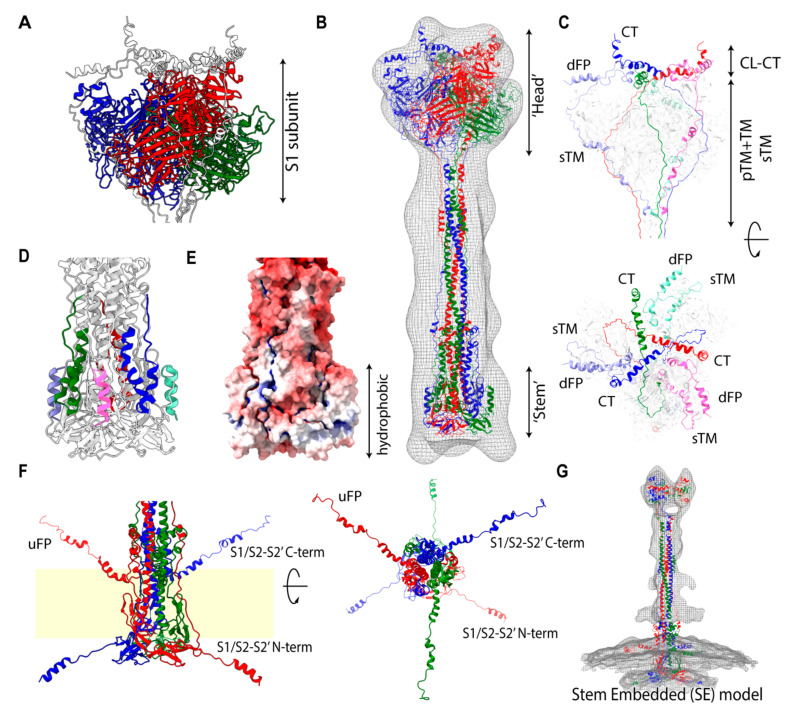
Computational model of the POST state structure. (**A**) A close-up of the previously uninterpreted region occupied by the S1 trimer. (**B**) Overview of the POST structure in solution fitted into the low-resolution Cryo-EM map. (**C**) A close-up of strands wrapping around the S1 trimer, with dFP + CL + sTM (light RGB) and pTM + TM + CT (RGB) shown. (**D**) Modeled fragments applied for disordered regions, N-term (light RGB), and C-term (RGB) of S1/S2-S2′ subunit. (**E**) Electrostatic potential of the stem (red: positive; blue: negative). (**F**) Both terms of S1/S2-S2′ are presumed to be extended. In the presence of the viral membrane, S1/S2-S2′ terms are presumed to extend along the surface of the membrane. A similar image was obtained using the Cryo-EM in moderate detergent. (**G**) Putative stem-embedded (SE) model fitted into the virion Cryo-ET map. The ‘Head’ region, having a large cavity, can be filled with transmembrane segments (TM, pTM, sTM), cytoplasmic segments (CT, CL), and dFP, but without the S1 subunit.

In the POST state in solution, spike proteins tend to aggregate and form a rosette-like multi-trimer [17]. The fusion peptide (FP), buried in the PRE state, is presumed to be exposed and gathered at the center of the rosette [4]. Although several POST state structures of SARS-CoV-1/2 have been resolved by Cryo-EM, all of them include large uninterpreted regions (Table S5). To model the full-length computational POST state structure, we employed a hybridization modeling approach to combine different parts of structures constructed by different computational modeling approaches (Materials and Methods). The low-resolution SARS-CoV-1 Cryo-EM map (resolution 30.5 Å, EMD-9597) was chosen as an overall shape constraint (Figure S3). The cytoplasmic (CL-CT) complex works as a ‘linchpin’, which provides strict constraints for extended transmembrane (eTM = TM + pTM) and sub-transmembrane (sTM) arrangements. The obtained computational model indicates that the uninterpreted region is likely occupied by the S1 trimer, while the cytoplasmic (CT-CL) complex in the S2′ subunit directly interacts with the S1s at the distal end of the uninterpreted region (Figure 3C). The CL forms a turn structure rolling into the adjacent dFP. Notably, the eTM and sTM are located at similar positions against a 3-fold axis wrapping around the S1 subunits (Figure 3A,C) with extended random-coil structures. Connecting the strands smoothly, eTM likely hooks the *β*-rich linker between NTD and INT1 of the S1 subunit, while sTM possibly hooks the linker between INT2 and CTD. Other disordered regions (Figure 3D) are fit into the hydrophobic stem region (Figure 3E). Other possibilities for structural arrangements for the eTM and sTM were denied due to the length restrictions or the hydrophobic–hydrophilic mismatches. The long C_α_–C_α_ distance between residues 779 and 919 (~141 Å, PDB: 6XRA) suggests that the POST state is likely realized after the S2′ cleavage. A part of the S1/S2-S2′ subunit being resolved in the atomic Cryo-EM structure [18] and a corresponding weaker band for S1/S2-S2′ in Western blot analysis [19,20] suggest an inter-subunit interaction between S2′ and S1/S2-S2′ as a complex in the POST state.

The computational structural model infers that the S1 trimer is still attached to S2 and wrapped by the eTM and sTM strands in the POST state. Indeed, the involvement of the S1 trimer in the POST state is supported by several observations. As mentioned, the S1 subunit is able to dissociate as a trimer in the PRE state [15]. The peptide screening study suggests that S1 is essential not only for the ACE2 receptor binding, but also for viral membrane fusion (Table S2). Furthermore, alanine scanning mutagenesis studies indicate that the L224A, L226A, I228A, T231A, and F233A mutations in SARS-CoV-1 NTD reduce viral entry (Table S2). These functional residues are located at the inter-monomer interfaces of the S1 trimer in the POST model, indirectly suggesting that the formation of the S1 trimer is essential for the membrane fusion (Figure S4).

### 2.5. Stem-Embedded Model Reveals a New Assignment into the POST-Like Density Map on the Virion

While the low-resolution SARS-CoV-1 POST spike density map was provided in the solution [17], the structures of SARS-CoV-2 spike were resolved under different experimental conditions, making comparisons challenging (Table S6). Here, we considered a few cases of POST structures interacting with a membrane (on the virion or with lipids condition).

To identify the protein regions interacting with the membrane in the POST state, we mapped the reported fusogenic segments, which were curated from the literature (Table S2), onto the POST structure (Figure S5) and assessed the membrane insertion free energy in the same manner (Figure S6). These results are reasonably consistent with each other. The fusogenic segments are localized at the stem, eTM, and sTM segments of the POST structures. These two fusogenic localized regions almost coincide with those obtained from the electrostatic analysis of polar–apolar regions (Figure S7), and indicate that the distance between two membranes at the POST state is approximately 215 Å. It is noteworthy that the stem, including the *β*-sheet rich region (BR), has been shown to interact with the membrane via peptide screening (Table S2). Hereafter, we considered the stem to be embedded into the viral membrane, then called the hypothetical model the stem-embedded (SE) model.

Chen et al. reported a high-resolution, post-fusion state SARS-CoV-2 spike structure (3.0 A, EMD-22293) and a variety of associated 3D structural classes in the POST state (Figure S8 in ref. [18]). One of the 3D classes shows multiple thin rods of density (at least 5 rods) extending radially from the stem. However, the Cryo-EM density map was not available for further analysis. Interestingly, the viral particles were processed to be soluble in detergent and the termini of the S1/S2-S2′ subunit (residue 686–702, 771–815) were disordered in the high-resolution structure (Figure 3D). The C-term region corresponds to the fusion peptide uFP and has a fusogenic activity (Figure 1A). To confirm that the radially distributed rods in the low resolution Cryo-EM map could correspond to the fragments of the spike protein, we further modeled the POST structure with the membrane by modifying the stem region of the POST structural model, also applying the force to pull the termini of the S1/S2-S2′ subunit. The obtained shape (Figure 3F) resembled one of the Cryo-EM 3D classes in the POST state in detergent (second left in the first classification of Figure S8 in [18]).

Furthermore, Li et al. reported on the Cryo-ET image in the POST state (15.3 Å, EMD-30428) anchored to the viral membrane [8]. However, the transmembrane densities in the virial membrane were totally missing for both the PRE and POST states. Based on the prior knowledge of the POST state modeling in this work, we modeled the POST-like extended structure, attempting to fit the density. The membrane position at the stem and the extended conformation of the S1/S2-S2′ subunit filled the missing density and the smaller volume of the head region was seamlessly fit by TMs, dFP, CT, and CL without the S1 subunit in the SE model (Figure 3G).

Despite having support, the SE model is still challenged since the assignment of the POST model in the original study was vertically upside down and the dFP and TM were anchored in the viral membrane. Hereafter, we call the conventional, widely accepted hypothetical assignment the FP-embedded model (FPE model). For a fair comparison, we analyzed the difference in fit between the SE and FPE models using the same Cryo-EM image and atomic structure, with the results shown in Figure S8. While the SE model presents a larger mismatched region around the viral membrane, the FPE model shows smaller regions around the ‘head’, certain glycans, and the viral membrane (Figure 3D in [21], Figure S8B). Although the volume of the mismatched region is larger in the SE model, this is feasible, as the PRE and POST densities obtained using Cryo-ET were mostly disordered above the membrane on the virion. In fact, the averaged 2D projection, i.e., the density images of the ‘head’ and ‘stem’ (Figure S2C in [21]), resembled that of SARS-CoV-1 (EMD-9597) [17], however the orientation was assigned as the FPE model in the [21], in which the uninterpreted region was fit by the stem of the POST structure. In other words, the assignment of the orientation to fit the ‘head’ and ‘stem’ images appeared to be inconsistent between [21] and [17]. Currently, no high-resolution Cryo-EM/ET map with the viral membrane is available that could unambiguously validate the orientation assignment directly.

Conventionally, the viral fusion steps mediated by the class I viral fusion protein have been widely accepted; therefore, many previous studies have followed the FPE model. However, this model also has its drawbacks and the features are hard to interpret as compared to the currently available experimental data. According to the fusion steps [22,23], the structural transitions from PRE to POST states are attained through the pre-hairpin and the fold-back states, where the dFPs of the spike protein interact with the host membrane. As a result, viral and host membranes are fused with the viral side (TM) and host side (dFP) of the transmembrane, meeting in the POST state (FPE model). Thus, the interaction between the dFPs and host membrane plays a crucial role in achieving the POST structure. Consequently, the feasibility of the FPE model on the virion in the absence of the host membrane is arguable.

### 2.6. Structural Comparison for the Transition from the PRE State toward the POST State

**Figure 4:**
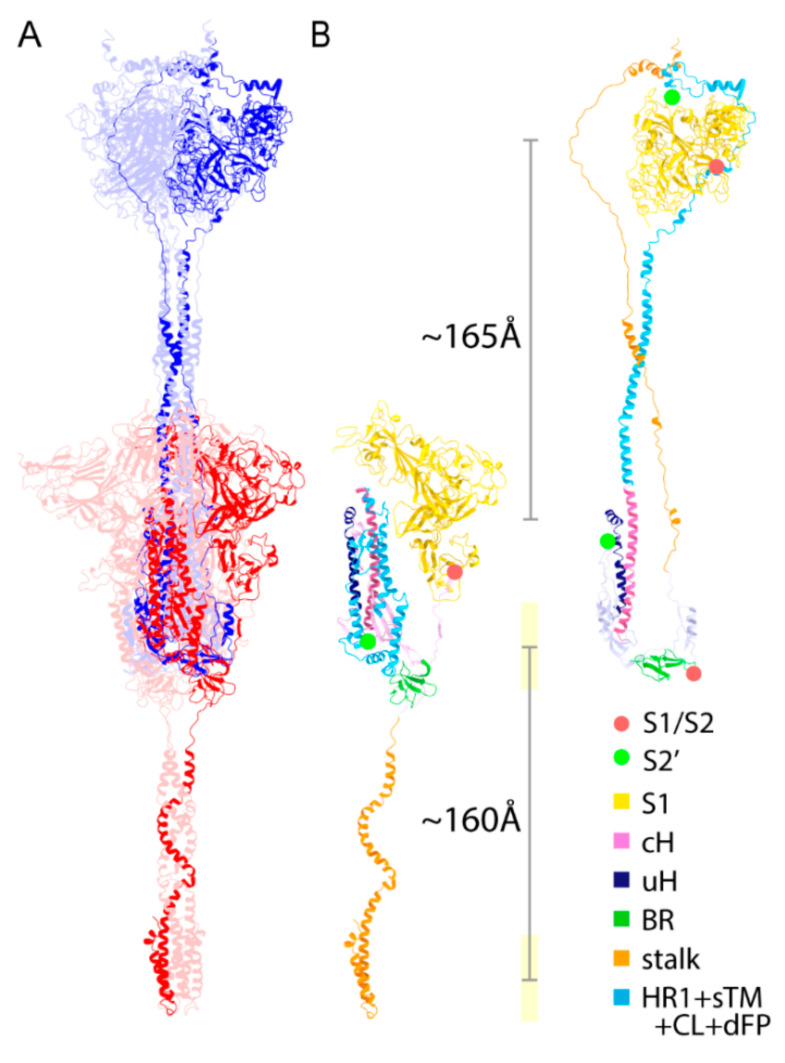
Structural comparison for a transition from the PRE state toward the POST state. (**A**) The structures of PRE (red) and POST (blue) states are shown. There are a few structurally maintained segments between PRE and POST states, including cH, BR, and uH. The figure is superimposed by the cH region. (**B**) A single monomer of the PRE and POST states (stalk: orange; BR: green; uH: dark blue; S1: yellow; cH: pink; HR1 + sTM + CL + dFP: light blue). The C_α_–C_α_ distances between residues 685 and 686 (red dots) and between 815 and 816 (green dots) are ~250 and ~240 Å, respectively. The distances of S1 centers and transmembrane regions between fitted PRE and POST structures are ~165 and ~160 Å, respectively.

As described in the previous structural study on SARS-CoV-1 [24], cH, BR, and a part of the S1/S2-S2′ subunit (uH) maintain their secondary structure in both the PRE and POST structures; however, the stalk in the PRE structure flips and forms an elongated random coil structure. The compactly folded HR1 also extends considerably during the structural transition toward the POST state, forming the inner core of the coiled-coil. In short, the stalk and N-term of S2′ (dFP + CL + sTM + HR1) are mainly responsible for the structural transition from the PRE state toward the POST state (Figure 4B). The structural transition includes the translocation of eTM and sTM, which are involved in wrapping around the S1 trimer in the POST structure to stabilize the S1 trimer attachment. To maintain the viral membrane position in the vicinity of the stem as presumed in the SE model (Figure S6), the precursor of the stem in the PRE structure has to translocate ~160 Å downwards. Similarly, the S1 trimer should translocate ~165 Å upwards, with a major conformational change (Figure 4B).

The cleavage sites S1/S2 and S2′ also translocate largely from PRE toward POST states. The C_α_–C_α_ distance for the S2′ cleavage (residue 815, 816) in the POST is ~240 Å, which suggests that a transition to the POST state is locked in before the S2′ cleavage, but may also imply the existence of another structural state between the PRE and POST states. For convenience, we introduce this intermediate state as the INT state, which is generated after the structural transition from the PRE state and before the S2′ cleavage. We also define an extended state (INT or POST state), as it is sometimes hard to distinguish between the INT and POST states.

Based on the obtained structural models and preceding experimental evidence, we propose a mechanism of the initial infection stage of SARS-CoV-2, including the viral membrane fusion.

### 2.7. Multiple Pathways Initiate the Viral Membrane Fusion after Binding to the Receptor

**Figure 5:**
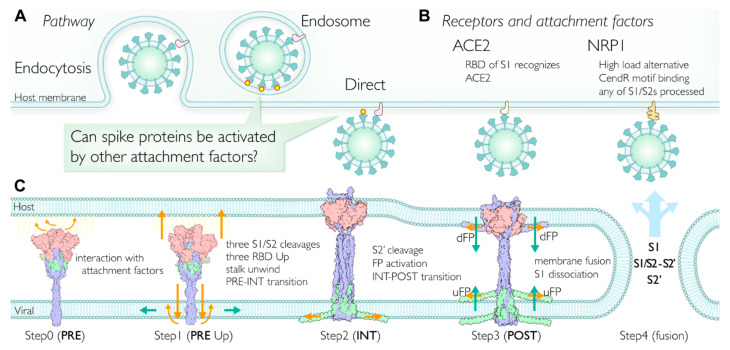
Proposed viral membrane fusion mechanism. (**A**) Two major pathways for cell entry. Direct entry is the primary pathway; however, endocytosis is also utilized. After the receptor binding, spikes may have more opportunities to facilitate the fusion steps by interacting with other attachment factors. (**B**) Typical receptors of SARS-CoV-2 for the membrane fusion. ACE2 is a primary receptor; however, the C-terminal of S1 subunit binds to neuropilin 1 (NRP1). The NRP1 pathway is considered to be utilized when the viral load is high. Note that the CendR motif binding to NRP1 is a unique insertion at the S1/S2 site in the SARS-CoV-2 spike. (**C**) The membrane fusion mechanism proposed in this study. Step 0 (PRE state): After binding a primary receptor such as ACE2, some spikes approach the surface of the host membrane. The RBDs are in thermal equilibrium, taking Up and Down forms. Soluble proteases such as furin can cleave the S1/S2 site in the spike during or before circulation (Table S8). Step 1 (PRE state RBD Up forms): A spike trimer approaches the host membrane surface and interacts with any attachment factors (Table S7) on the membrane surface, leading to the equilibrium shift toward the RBD Up form. Step 2 (INT state): The intermediate (INT) state between PRE and POST states before the S2′ cleavage. A structural transition to INT is triggered by the conditions (RBD Up forms, S1/S2 cleavages). When the two conditions are satisfied, subsequently the unwinding of the stalk occurs, which unlocks the structural transition (untwisting activation mechanism). As a result of the structural transition from PRE to INT, the stalk in S2′ unfolds toward the host membrane and wraps around the S1 trimer. Concurrently, the stem integrates into the viral membrane and N-terms of S1/S2-S2′ expand onto the inner surface of the viral membrane to stabilize the stem. The S1 trimer is facing against the host membrane surface, and eventually the S1 trimer integrates into the host membrane. Step 3 (POST state): The S2′ cleavage event activates two pieces of fusion peptides (uFP and dFP) located at the S2′ cleavage site and triggers membrane fusion. In concert with the N-term of S1/S2-S2′, uFP and dFP moderate and facilitate the curvature of the membrane. Step 4: Viral and host membranes are fused. S1 subunits dissociate and are shed into the host cell. The rest of the proteolytically processed spikes, namely the S1/S2-S2′ and S2′ subunits, are possibly disassembled and delivered into the host cell as well (PRE, INT, and POST structures are colored by subunits).

To date, the plasma membrane (direct entry) [25] and endosomal [26] viral fusion pathways have been reported for SARS-CoV-2 entry into cells (Figure 5A). An RBD of the spike recognizes an ACE2 as a primary receptor. Although several studies have concluded that ACE2 and the S1 subunit complex dissociate together and initiate the infection, certain conditions (lower pH environment [11], lower concentration of ACE2 [27], conformational instability [28]) support an initiation of ACE2 dissociation from S1. Regardless, the interaction between the ACE2 and S1 RBD has a primary role in initiating the infection process (Figure 5B).

Once one of the S1s recognizes the primary receptor (ACE2), there is a higher probability that other spike proteins will start interacting with the host membrane directly and that the membrane fusion steps will be initiated (Figure 5A). The spike proteins are also thought to interact with other attachment factors such as lipids, glycans, and transmembranes (Table S7). The involvement of lipid rafts has been reported in some cell cultures for coronavirus entry [29,30]. The host attachment factors likely interact with known or putative binding sites at the spike RBD, which is required to take the RBD Up form.

### 2.8. Involvment of S1/S2 Cleavage and Untwisting Activation Mechanism in the Structural Transition

The POST state is thought to be a lower free energy state than the PRE state, while the free energy barrier for its structural transition to the extended state is also low [18,26]. The S1/S2 cleavage by proteases (Table S8) is presumed to initiate the structural transition [31]. When the S1 trimer interacts with the host attachment factors at the membrane surface, S1 RBDs likely maintain their interactions with the host factors by taking the Up forms, while only S2 ectodomain rotates after the S1/S2 cleavages. Simultaneously, the S2 ectodomain rotation unwinds the stalk trimer and disassembles the extended transmembrane (eTM) into monomers, which triggers the stalk transition and the larger structural transition to the extended state sequentially (Figure 2D and Figure 4B). Thus, this step could be the mechanism behind the wait for the three RBD Up forms, which utilizes the thermal fluctuation (untwisting activation mechanism). The conditions required for the equilibration shift from winding to unwinding of the stalk are (1) three S1/S2 cleavages and (2) three RBD Up forms. This equilibration shift is possibly a rate-limiting step in the PRE state; however, the subsequent structural transition appears to be an irreversible process [18,31]. This view coincides with the NMR studies. After the equilibrium of the parallel HR1-HR2 between the structured HR2 (winding) and unstructured monomer HR2 (unwinding), the irreversible transition to antiparallel HR1-HR2 was observed [32,33]. Note that the trimeric coiled-coil structure in the stalk is assembled with an inter-monomer hydrophilic interaction. The disassembly of the flexible HR2 trimer to unwind the stalk also requires the compensative free energy gain. The SE model with the membrane-anchored stem accounts for the compensation. We speculate that the insertion of the stem into the membrane also provides a free energy gain, so that TM regions overcome the free energy barrier to be released from the viral membrane.

It is also noteworthy that the S1/S2 subunit can be processed before recognizing the receptor [34,35]. Although the appearance of three RBD Up forms of the spike protein appears to be a rare event, a mutagenesis structural study on SARS-CoV-1 showed that the spike protein stabilized in the PRE state exhibits all RBD Up forms (3%) without receptor binding [36]. The POST-like extended spike on the virion can also be explained using the SE model.

### 2.9. Extended Transmembrane (eTM) Wraps around a Trimeric S1 and Subsequently a Long Internal Core (LIC) Forms a Long Coiled-Coil toward the POST State

The obtained POST model structure suggests that the S1 subunit can be present as a trimer and that eTM and sTM can wrap around the S1 trimer in the solution (Figure 3A–C). PRE ectodomain structures in other coronaviruses suggest that a large opening motion of S2, exposing the hydrophobic interface, drives the transition from PRE to POST states [37], whereby the stalk has to unwind and disassemble to enable the opening motion (Figure 2D and Figure 4B). If LIC moves earlier than the stalk, a pivot motion cannot occur and the dislocation of sTM + HR1 upwards leaves an empty space, which would likely lead to the collapse of the spike structure (Figure S10). On the other hand, some studies indicate an association of HR1 trimer forms prior to HR2 [31]. In summary, the eTM wraps around the S1 trimer and subsequently the LIC forms a long coiled-coil.

### 2.10. Role of Intermediate (INT) State Realized after the Transition before the S2′ Cleavage

Although it is not apparent, the cleavage site S2′ is occluded in the PRE state [36]. This suggests that the S2′ cleavage is only feasible after the S1/S2 cleavage [19], and that the S2′ site likely becomes accessible in the extended state after the structural transition (Figure 4B). Additionally, the distance constraint between residues 815 and 816 at the S2′ cleavage site from the POST structure requires the intermediate (INT) state. Following the discussion of eTM and LIC conformational changes, the INT state may be reached after eTM wraps around an S1 trimer. Note that the S1 wrapping by sTM, CL, and dFP that occurs in the POST state is not feasible due to the restriction of the uncleaved S2′ in the INT state.

### 2.11. Initiation of Membrane Fusion and S1 Trimer Release

Upon the cleavage of S2′, the long continuous FP is divided into two terminal FPs, uFP and dFP, and these FPs are activated in the sense that free FPs directly interact with the membrane. According to the structural requirements, uFP and dFP should mainly interact with the viral and host membranes, respectively. As seen in the extended conformation models of the uFP in the viral membrane (Figure 3F,G), the NMR study also suggests the extension of dFP in the host membrane [38,39] in cooperation with CL [40]. Once the extension of uFP occurs, we speculate that eTM cannot hold the S1 trimers, which results in the S1 release. Consistently, biochemical experiments have exhibited the distinctive spontaneous S1 dissociation state after the S1/S2 cleavage [41], implicating the detailed timing of the S1 dissociation in this step. Importantly, free S1 is permeable across the lipid membrane without interacting with receptors [42]. Thus, a release of the S1 trimer into the membrane likely triggers distortion and instability of the membrane surface. The interactions of dFP and sTM with the membrane are also suggested to induce the order and curvature of the host membrane [43,44]. S1/S2-S2′ subunits are likely present as parts of the spike protein complex [18,19], and play important roles in stabilizing viral membrane binding through the INT–POST state transition, facilitating the membrane fusion. It is not clear how many S2′ cleavages are required for the S1 dissociation.

Considering the role of S1 on the host membrane, the release of S1 subunits from the POST spikes [18] may be regarded as inactivation of spike proteins; in other words, the loss of the membrane fusion capability. In some studies, the importance of the S1 dissociation has been discussed in the context of infectivity [42,45].

### 2.12. Viral Membrane Fusion Mechanism: Proteolytic Cleavage Events, Distinct States, and Membrane Fusion

We realized that the PRE and POST did not simply refer to ‘fusion’, but rather to the ‘structural transition’ states. Although proteolytic cleavages in spikes appear to be essential for the infection [46,47], alternative pathways, alternative receptors/attachment factors (Table S7), alternative proteases for S1/S2 cleavage (Table S8) exist. Although the primary protease for the S2′ cleavage is TMPRSS2, which is responsible for the plasma membrane pathway, cathepsin L appears to be the alternative protease for the endosomal pathway. However, it is not clear whether cathepsin L cleaves the S2′ site or not. Several studies indicate the essential role of cathepsin L in TMPRSS2-deficient cells [48,49]. Single-cell transcriptome profiling analysis suggests that the brain, esophagus, and heart are TMPRSS2-deficient tissues, while the brain, esophagus, lungs, liver, and prostate are ACE2-deficient tissues [50]. The tropism at the S1/S2 site mutations in the absence of TMPRSS2 implies that the CendR motif is a consequence of the highly optimized S1/S2 site in SARS-CoV-2 through evolution of the TMPRSS2 pathway [48,51]. SARS-CoV-2 appears to have a fitness advantages in various environments. Figure 5B summarizes our proposed model discussed above.

### 2.13. Comparison with Conventional Steps Mediated by Viral Class I Fusion Protein

The proposed viral membrane fusion mechanism differs from the traditional steps mediated by class I viral fusion proteins [22,23], consisting of pre-fusion, pre-hairpin, fold-back, and post-fusion states. To date, several studies have highlighted the direct visualization of pre-hairpin states and few highlighted the fold-back state. Although it is possible to distinguish between the conventional pre-hairpin state and our extended state (INT and POST states) by measuring the width between two membranes, and while several studies appear to support our model, there are still contradictory images. Additionally, it is possible to validate the model by capturing the distributions (existence) of spike proteins at the concave virus–host cell interface. Following our mechanism, the spikes should be located close to the center of the tangent region and possibly forming an ‘entry claw’ similar to the one from the HIV-1 entry [52] to create multiple small pores initiated by the S1 integration; however, we leave the arguable discussions based on the direct visualizations here. Further striking images are awaited.

Other than the direct observations, a kinetics study using pulse-labeling hydrogen–deuterium exchange mass spectrometry and Cryo-ET suggested no observable hairpin or fold-back states during the transition from PRE to POST states in one of the class I fusion proteins (influenza virus hemagglutinin) [53]. The critical function of the stalk for cell–cell infectivity in SARS-CoV-1 implies that the eTM is responsible for the pore formation and the enlargement during the fusion [54]. Parts of the S1 and the stem are able to induce membrane leakage in the peptide screening, suggesting their direct role in the membrane fusion [55]. Our model accounts for these experimental studies. A detailed comparison is given in Table S10. Our model shows that the membrane fusion can provide effective means to deliver some of the proteolytically processed spike proteins (namely the S1, S1/S2-S2′, S2, S2′) and the viral RNA through the host membrane.

### 2.14. Case Studies

Here, we present two examples of spike protein characteristics associated with the antibody binding to the spike protein and the prevalent SARS-CoV-2 spike variants, filling gaps in our previous understanding.

### 2.15. Case Study 1: Effect of Neutralizing Antibody Binding to the Spike Protein

Neutralizing antibodies defend cells from the virus, and more than 15 therapeutic antibodies for the COVID-19 treatment are reported to be in preclinical development or in clinical trials [56]. There are several neutralizing strategies, with the RBDs of S1 as being promising targets [57]. S230 and LCA60 are two potent human neutralizing antibodies that have been shown to be effective against SARS-CoV-1 and MERS-CoV, respectively. In the structural studies of coronavirus antibodies, only S230 has been shown to allow the structural transition from the pre-fusion state to the post-fusion state, in contrast to LCA60 [58]. According to the untwisting activation mechanism, three S1/S2 cleavages and three RBD Up forms are the determinants for the structural transition. As expected, antibody-binding Cryo-EM structures and statistics showed that S230 only interacts with Up or intermediate RBD forms, while LCA60 can recognize all of Up and Down forms (one Down ~50% and two Down ~50%). It is also noteworthy that the structural transition to the extended state is reported to involve the presence of trypsin, which cleaves the S1/S2 site but not the S2′ site of SARS-CoV-1; hence, cleavage of S1/S2 but not S2′ is responsible for the structural transition to the INT state.

### 2.16. Case Study 2: D614G Variant Has High Infectivity and Transmissibility of COVID-19

The high binding affinity of the spike protein to the ACE2 receptor is one of the defining factors that explain the high cell infectivity of SARS-CoV-2 relative to SARS-CoV-1 [59]. Although transmissibility and infectiousness are not always synonymous, more transmissive variants normally become more prevalent in the population via natural selection [60,61]. To date, there are several mutations of the SARS-CoV-2 spike protein, including N439K [62], Y453F, and N501Y [63], which are located at the interface between the S1 RBD and ACE2, having increased binding affinity to ACE2. However, some variants, for example D614G, have slightly lower ACE2 affinity [13,64] but higher viral pathogenesis and transmissibility [61], while having no notable difference in neutralized antibody sensitivity [65]. Despite the fact that the D614G variant has been prevalent during the pandemic [60], exhibiting higher viral load and replication [65], the advantage of the D614G variant in terms of the infection mechanism is still unclear.

Significantly, the D614 in the CTD of the S1 peptide interacts with CL, which works as a switch for the transition of the Up and Down forms (Figure 2B). D614 hydrogen bonds to I834 (backbone), K835, Y837, K854, and T859, altering the conformation of dFP and CL [11,13]. In a comparison between G614 and D614 residues, the D614 residue acts as a ‘latch’, while the D614G variant loses the interaction with CL, leading to a more frequent RBD Up form [13]. A shift to the frequent Up form likely lowers the transition free energy barrier and results in higher infectivity. Consistently, the transition barrier of SARS-CoV-2 is also suggested to be lower than that of SARS-CoV-1 [26].

Notably, the D614G variant has been reported to show less S1 shedding but higher incorporation of infectivity into the virion [64]. The weaker interaction at the S1–S2 interface or a higher structural transition rate to the extended state does not explain these two aspects. Following our model, the S1 trimer shedding occurs after the second cleavage at the S2′ site in the presence of TMPRSS2. The CL and dFP work concertedly to hold the S1 trimer, while the S1 trimer creates a pore on the host membrane surface [42]. We suspect that the G614 residue weakens the interaction with CL in the PRE state so that CL can wrap around the S1 trimer more efficiently during or after the structural transition, which is consistent with the increased S1 trimer association in the D614G variant [66]; hence, the D614G variant has synergic advantages in the infection steps as a whole. Although the more frequent RBD Up form suggests a higher likelihood of receptor binding, it is important to emphasize that the aforementioned experiments implicate additional roles of the S1 subunit in the infection after the receptor binding.

## 3. Concluding Remark

We have reported on computational structural models of the full-length SARS-CoV-2 spike and proposed a novel viral membrane fusion mechanism. The fusion model accounts for most of the experimental studies. The previous fragmented knowledge should be carefully validated and selected, considering the implications of the experimental conditions, in order to provide a comprehensive view.

Our computational structural models were developed using a low-resolution Cryo-EM map and constructed by assembling predicted structural fragments as the best approach. Further refinements of the models should be considered to discuss structural details at the atomic level. Preferably, high-resolution Cryo-EM density maps in INT and POST states are awaited. Further developments of structure–function models of the spike protein interactions with the host membrane are likely to lead to additional targeted antibody and drug therapies that will reduce the infectivity of the virus and account for altered infectives in novel and emerging variants.

## 4. Materials and Methods

### 4.1. Modeling of the PRE State

Step 1. Construction of a stalk in the PRE state. To validate the twisted coiled-coil structure, we modeled a trimer structure of a stalk in the PRE state without applying any twisting bias. Considering the disordered region, we modeled the last 140 residues at the C-term of the PRE spike, constructing 40 to 140 residue fragments (Figure S9). *Step 1-A.* Six 40 residue *α*-helix fragments with ideal parameters were prepared. *Step 1-B.* A Rosetta symmetric docking protocol was used to generate trimers for each 40 residue α-helix fragments. *Step 1-C.* The 20 residue trimers were extended to 40, 60, 80, 100, 120, and 140 fragments using a Rosetta hybridization protocol. As the templates, the trimer fragments in step 2 were used. Additionally, the top 3 scoring structures from the shorter fragment simulations were selected as templates. However, structures with positive scores were discarded from the template. For step 1-C, the distance constraints were applied on C_α_ atoms onto residues 1140 and 1144 to fix the upstream side of the stalk. A Cryo-EM structure (PDB: 6XR8) was used as the constraint reference.

Step 2. Construction of a full-length PRE state structure. To construct a full-length PRE spike structure, the Cryo-EM structure (PDB: 6XR8) and ab initio stalk structure used in the previous step were used as the templates in the standard Rosetta hybridization protocol. We removed the density around the stalk from the original Cryo-EM map (EMD-22292) (Figure S11) and used it as the constraint.

### 4.2. Modeling of POST State

#### Modeling Strategy

To obtain the full-length spike protein structure in the POST state, we used a low-resolution Cryo-EM density map obtained from the SARS-CoV-1 spike (EMD-9597, resolution: 30.5 Å) and the atomic structure of SARS-CoV-1/2 spikes (PDB: 6M3W, 6XRA). The Cryo-EM density has a large uninterpreted volume, which is almost C3 symmetric, although is not exactly evenly shaped. Although the low-resolution density map was obtained with the ectodomain, we assumed that the low-resolution density map designates the overall shape. Having identified the overall shape, we adopted a divide and conquer strategy. We initially modeled the small parts and then assembled them with a standard Rosetta hybridization protocol. From the size and shape of the uninterpreted region (Figure S3), the S1 trimer likely fit into the volume. The rest of the missing components were fusogenic segments (dFP, CL, sTM, pTM, TM, CT). Considering the fusogenic properties, hydrophobic segments (sTM, eTM) should form a compact structure in the solution. The relatively hydrophilic segments (CL, CT) should be allocated at the distal end of the uninterpreted volume, where the cytoplasmic region is supposed to be located if the membrane is present.

Step 1. Modeling of the S1 trimer using flexible fitting. To explore the possibility that the uninterpreted volume is attributed to the S1 trimer, we performed flexible fitting using molecular dynamics (MD) simulation. The NAMD program with the Molecular Dynamics Flexible Fitting (MDFF) protocol was used, applying a C3 symmetric constraint in the explicit solvent environment. Step 2. Modeling of dFP, CL, and CT complexes. The ‘linchpin’ of the POST structure in solution is the meeting point of CL and CT. Initially, we obtained the docked structures of CL (residue: 838–854) and CT (residue: 1235–1273) with the Rosetta docking protocol (Figure S12A). Using the optimized monomeric CL–CT docking structures as the input, an optimal trimeric structure of the CL–CT complex was obtained by applying a C3 symmetric constraint (Figure S12B). The structures, which did not fit the Cryo-EM density, were excluded from the candidates. Step 3. Modeling of N-term of S1/S2-S2′ subunit and upstream of uFP. The fragments of the disordered region were obtained ab initio. The TrRosetta and Ab-initio Relax protocols were used for the structural modeling of the N-term of S1/S2-S2′ (residue id: 667–705) and C-term of S1/S2-S2′ (residue id: 797–815), respectively. Step 4. Modeling of sTM and eTM. Both sides of sTM and eTM strands were determined by the CL–CT complex in step 2 and the Cryo-EM atomic structure. Therefore, there were not many options to connect the strands of sTM and eTM smoothly. The strands were manually placed to connect the CL–CT complex and the Cryo-EM structure and were relaxed using NAMD. Step 5. Assembly of the parts. The obtained fragments were used as templates and assembled with a standard Rosetta hybridization protocol. The Cryo-EM density map (EMD-9597) and C3 symmetric constraints were applied.

For step 4, we considered other possibilities of the CL–CT complex positions and sTM and eTM strand connections, such as the possibility that the sTM strand or helix is structured along the 3-fold axis inside the S1 trimer. However, the possibilities were denied because of the length restriction or inconsistency of the hydrophobic–hydrophilic properties. A list of the software used in this work and a summary of the resolution and sampling statistics for the modeling are provided in Tables S11 and S12, respectively.

## Supplementary Materials

The following are available online at https://www.mdpi.com/article/10.3390/v13061126/s1, Figure S1. Prediction of the transmembrane region, Figure S2. Sequence alignment between SARS-CoV-1 and SARS-CoV-2, Figure S3. Cryo-EM density used for the constraint for the POST structure modeling, Figure S4. Functionally important residues in S1, Figure S5. Fusogenic segments mapped on the PRE and POST structures, Figure S6. Free energy for membrane insertion mapped onto the PRE and POST structures, Figure S7. Electrostatic potential on the surface of PRE and POST structures and presumable viral and host membrane positions in SE model., Figure S8. Comparison of the POST structure orientations on the virion., Figure S9. Steps to construct a stalk in PRE state, Figure S10. Hypothetical dislocation of the sTM+HR1 segments, Figure S11. Cryo-EM density used for the constraint in the PRE structure modeling, Figure S12. Optimized POST state structure in the cytoplasmic region, Table S1. Abbreviations used in this study, Table S2. List of segments with previously reported functionality (fusogenic activity) in the literature, Table S3. List of the publicly available spike protein computational models, Table S4. Examples of rotational motion of an S1 trimer, Table S5. List of deposited EMDB density maps and PDB structures, Table S6. Summary of the population in extended (INT/POST) state on the SARS-CoV-2 virion, Table S7. List of putative host factors including receptors and attachment factors, Table S8. List of enzymes reported with the cleavage sites, Table S9. Summary of experimental conditions that are successful in obtaining atomic SARS-CoV-1/2 spike structure in POST state in literature, Table S10. Comparison of viral fusion mechanism between our model and the conventional model mediated by class I fusion protein. The limited items are listed to compare, Table S11. List of software used in this study, Table S12. Resolution and sampling statistics for the original domains.

## Data and Materials Availability

All data needed to form the conclusions in the paper are presented in the paper or the Supplementary Materials.
