# Supplementary material for: Full-Length Computational Model of the SARS-CoV-2 Spike Protein and Its Implications for a Viral Membrane Fusion Mechanism
Source: Viruses. 2021 Jun 11;13(6):1126. doi: 10.3390/v13061126 (PMC8230804; doi:10.3390/v13061126)
Supplement: Supplementary file 1 [file viruses-13-01126-s001.zip › viruses-1232484-SI.pdf]

viruses

MDPI

Supplementary material for:

Full-length computational model of the SARS-CoV-2 spike protein and its implications for a viral membrane fusion mechanism

Wataru Nishima [1,2,*] and Marta Kulik [3]

1 New Mexico Consortium, Los Alamos, NM 87545, USA
2 University of New Mexico, Albuquerque, NM 87131, USA
3 Biological and Chemical Research Centre, Department of Chemistry, University of Warsaw, Żwirki i Wigury 101, 02-839 Warsaw, Poland
* Correspondence: wataru.nishima@gmail.com

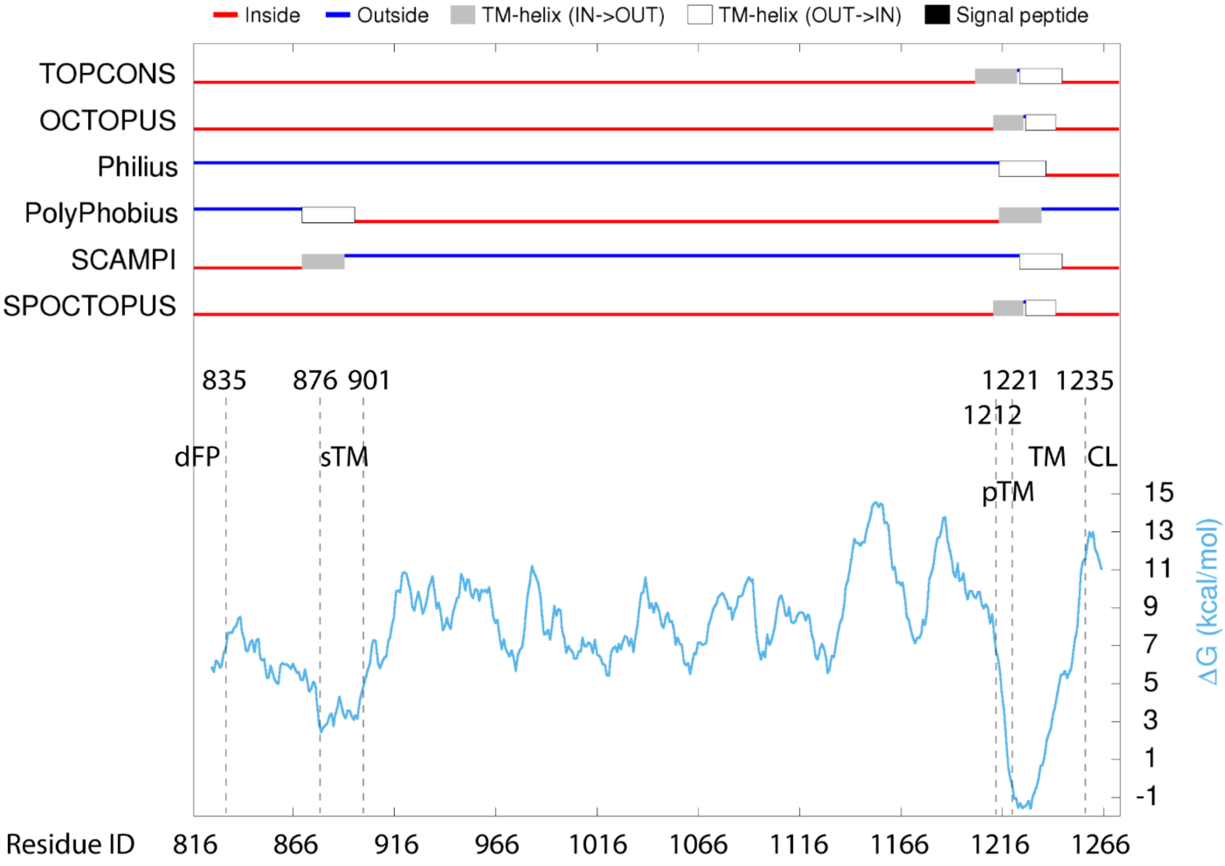

Figure S1. Prediction of the transmembrane region. The topology of membrane protein was predicted using TOPCONS [1], which performs multiple transmembrane prediction programs including OCTOPUS, Philius, PolyPhobius, SCAMI, SPOCTOPUS. These programs utilize amino acid sequence based prediction algorithms. The free energy profile of the S2' subunit for the membrane insertion with a window of 21 amino acid segment centered around each position is shown. The sTM, pTM+TM region have lower free energy. The pTM+TM is predicted as a transmembrane by all of the prediction programs. sTM is also predicted as a transmembrane by the PolyPhobius and SCAMPI. It is noteworthy that we allocate the cytoplasm region (CL) between dFP and sTM, which are relatively hydrophilic.

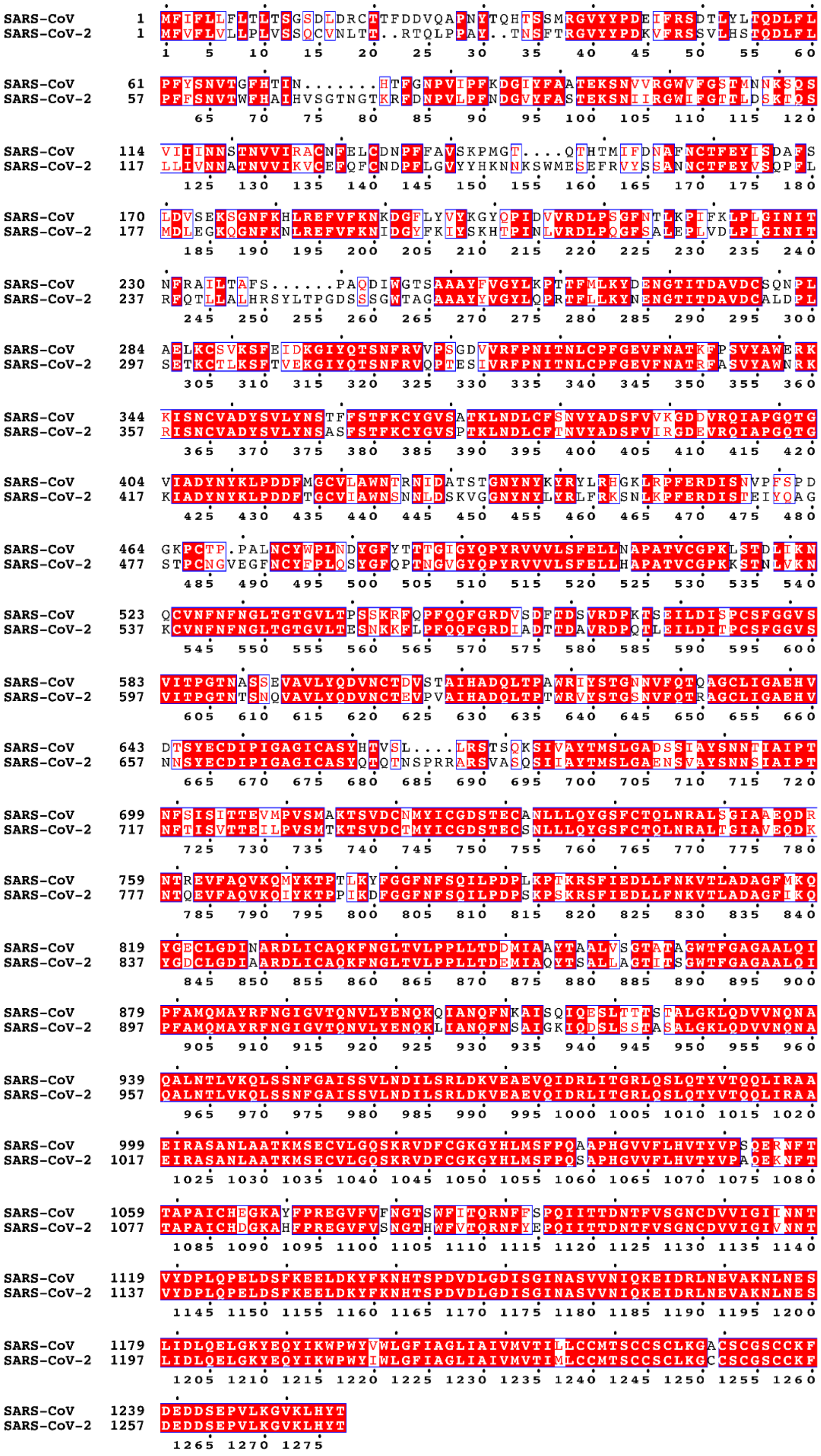

Figure S2. Sequence alignment between SARS-CoV-1 and SARS-CoV-2. The sequences of SARS-CoV-1/2 are obtained from Uniprot (P59594, P0DTC2). The sequence alignment was conducted using MAFFT [2]. The result was visualized using ESPript [3].

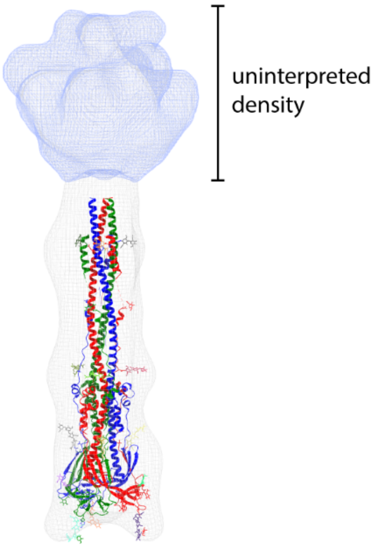

Figure S3. Cryo-EM density used for the constraint for the POST structure modeling. SARS-CoV-2 spike protein structure in POST state (PDB: 6XRA) was fitted into the Cryo-EM density map obtained for SARS-CoV-1 [4] (EMD-9597, resolution: 30.5 Å). There is large uninterpreted density region in the 'Head' region. The blue mesh volume is approximately 518,000 $Å^3$ (contour level: 0.033).

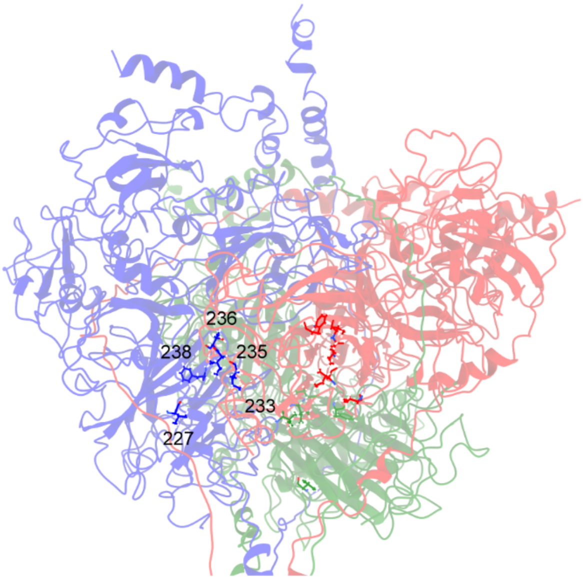

Figure S4. Functionally important residues in S1. Mutagenesis studies of the SARS-CoV-1 suggest L224A, L226A, I228A, T231A, and F233A reduces the viral entry. The corresponding residues for the SARS-CoV-2 are I227, I233, I235, T236, F238. I227 is interacting with the pTM strand. I233, I235, T236, F238 are localized near the inter-monomer interface. The mapping result implicates that the formation of an S1 trimer is important for viral entry.

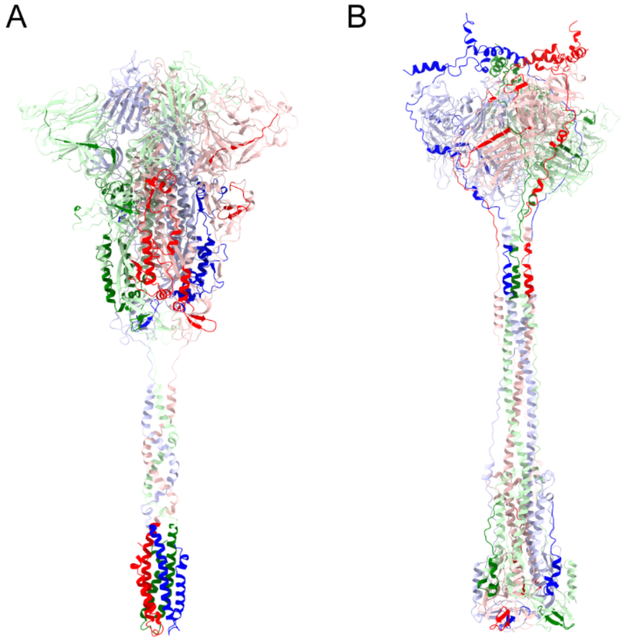

Figure S5. Fusogenic segments mapped on the PRE and POST structures. (**A**, **B**) PRE (A) and POST (B) structures colored by fusogenic segments (RGB) reported in the literature (Table S2). Both terminals of S2 (uFT, CT) involving the large structural transition from PRE to POST(INT) have fusogenic activities, and a large portion of stalk as well as fusion peptide related segments (N-term of S2) wraps the S1 trimer. uFP, as well as BR in POST structure, are located at the stem. In our proposed model, these fusogenic segments in the POST structure are extended along the surface of viral and host membranes.

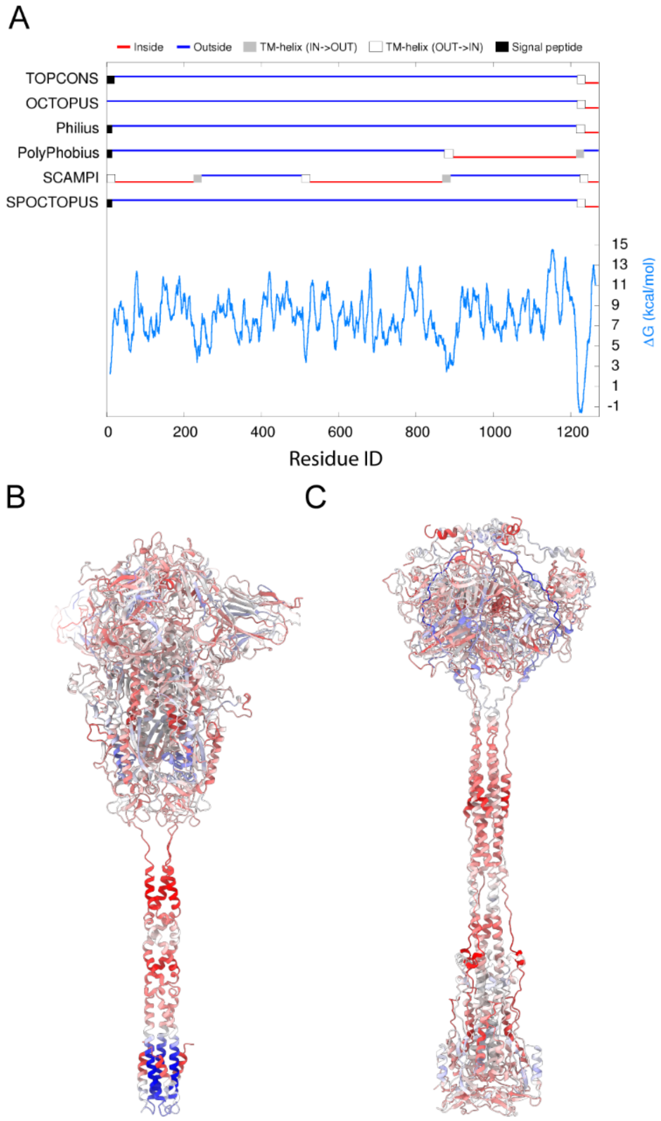

Figure S6. Free energy for membrane insertion mapped onto the PRE and POST structures. (**A**) Free energy profile for the membrane insertion obtained by TOPCONS [1]. The program predicts the free energy from the amino acid sequence. Note that SCAMPI predicted two regions in S1 as transmembrane, which implicates the S1 can be embedded with the membrane. (**B**, **C**) The color mapping of membrane insertion free energy for the PRE (B) and POST (C) structures, respectively. The first and last 9 residues are truncated due to the no data for the membrane insertion free energy. Interestingly, the high and low values of free energy are densely located at the stalk in the PRE state. However, some of the large mismatches are observed in NTD, RDB, sTM of the PRE ectodomain (B). On the other hand, the corresponding eTM region is extremely elongated as the random coil in POST state. The mismatched signal is more optimized in the POST structure and creates two bands near the stem and S1 regions (C).

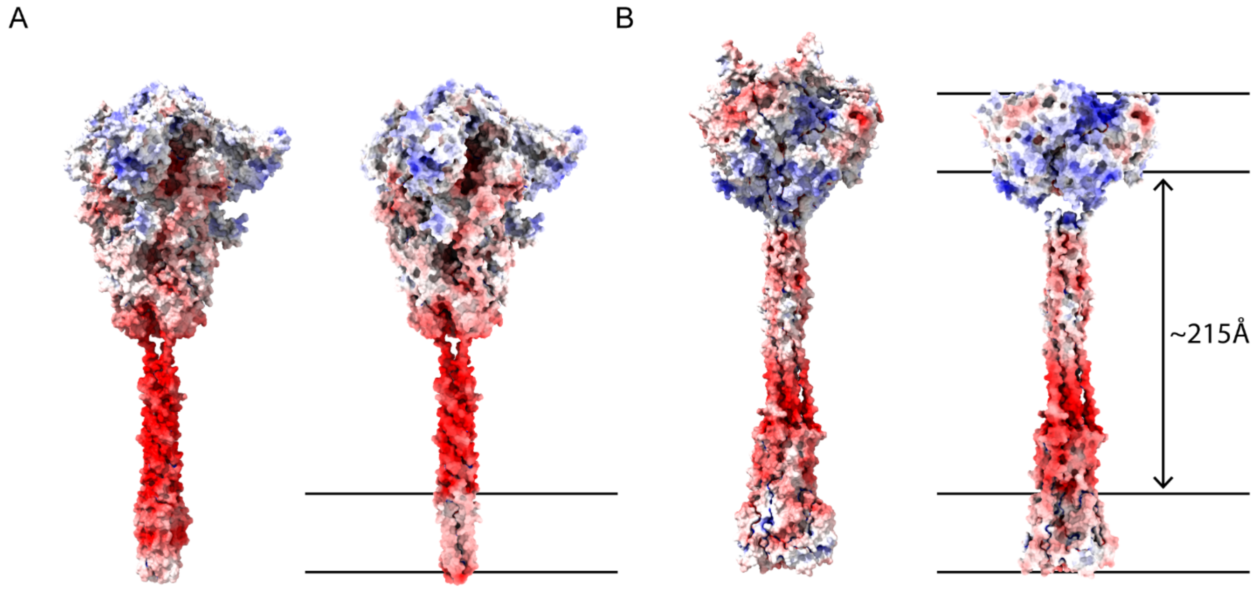

Figure S7. Electrostatic potential on the surface of PRE and POST structures and presumable viral and host membrane positions in SE model. (**A**, **B**) Electrostatic surface potential of PRE (A) and POST (B) structures are colored by red and blue for negative and positive values. Presumable positions of viral- and host- membranes are the regions between solid lines. Note that the full-length structures and electrostatic potentials for both full-length and truncated structures are calculated in the solution environment using APBS [5] and PDB2PQR [6]. The left figures of each panel A, B are made with full-length structures, while the right figures are made with structures truncating mobile segments at fusogenic localized regions (residue ids 1241-1273, and ids 686-719, 771-906, 1207-1273 were truncated for the PRE and POST, respectively). In the SE model, the CTs of the PRE structures are considered to expand along the inner surface of the viral membrane, while both terms of the S1/S2-S2′ subunit extend from the stem region (Fig. 3F) and the main fusion peptide (dFP) and sTM are also considered to extend in the host membrane. With this assignment, the distance between two membrane surfaces is approximately 215 Å.

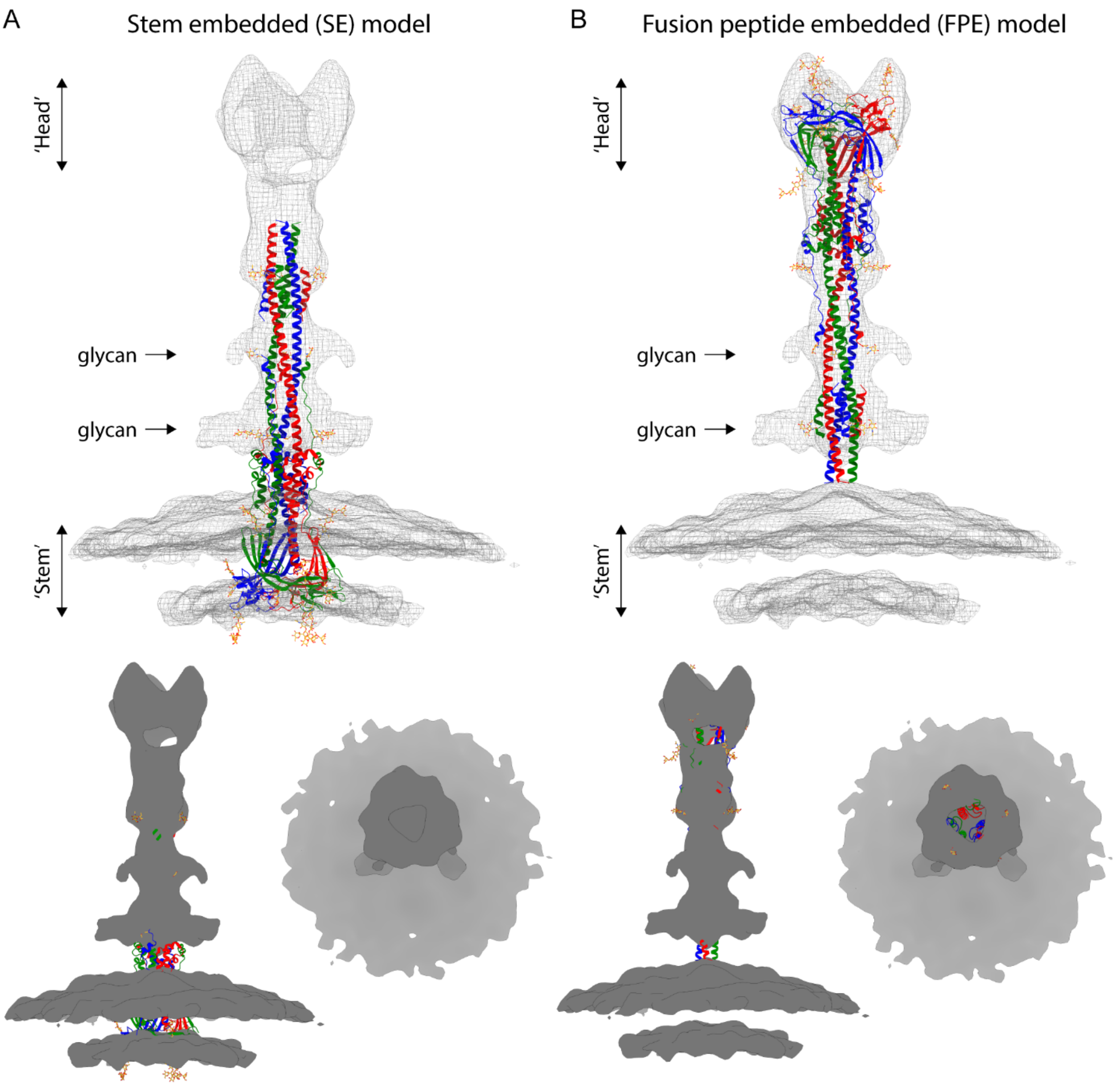

Figure S8. Comparison of the POST structure orientations on the virion. (**A, B**) Two structural orientations to fit the Cryo-ET image of the extended state on the virion (EMD-30428) are shown. The 'Head' region of the Cryo-ET image has a large cavity. For the fair comparison, the same SARS-CoV-2 POST structure (PDB: 6XRA) was used for the fitting. The glycans positions were also maintained as in the Cryo-EM structure. (A) Stem Embedded (SE) model. The stem is embedded in the viral membrane. The disordered segments, dFP, sTM, pTM, TM, are located at the top of the extended density. We support the SE model. (B) Fusion Peptide Embedded (FPE) model. Disordered segments, dFP, sTM, TM, are located in the viral membrane. In the main text, further the modeling including complete S1/S2-S2′, S2′ subunits are described (Fig. 3G).

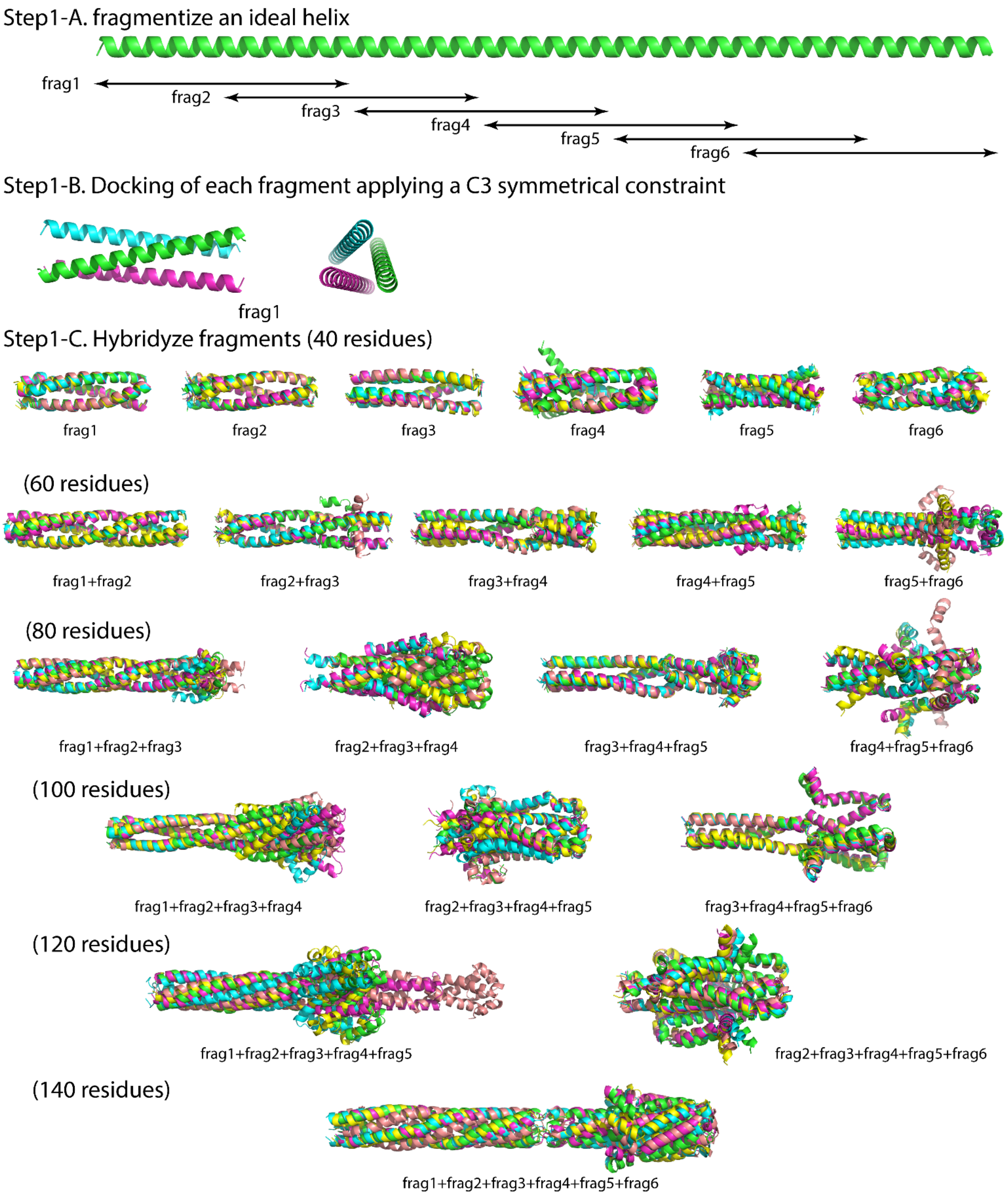

Figure S9. Steps to construct a stalk in PRE state. Rosetta hybridization protocol was iteratively used, starting from shorter fragments (40 residues) to longer fragments (140 residues). The top 5 structures are shown in Step1-C. Note some of fragments (e.g., frag4, frag6) tend to fold and form compact structures (e.g., frag2+frag3+frag4+frag+5+frag6), suggesting the stalk twist is optimal as the 140-residue trimer with the N-term constraint. It appears the coiled-coil structure is out of the global minima without the constraint.

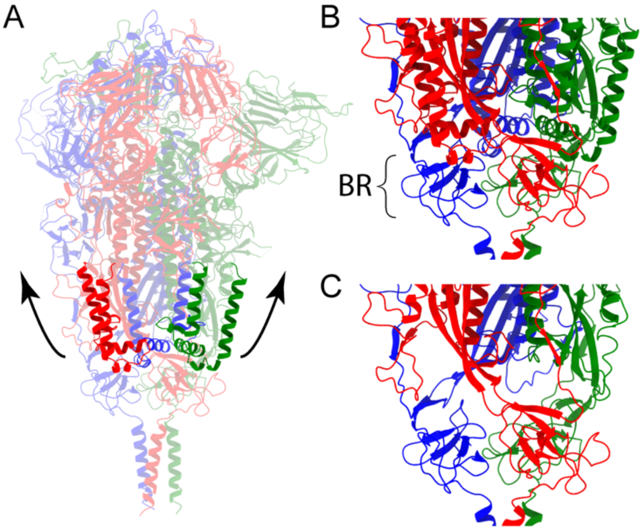

Figure S10. Hypothetical dislocation of the sTM+HR1 segments. (**A**) the position of the sTM+HR1 fragments (residue 866-942) in PRE state. (**B**) In the PRE state, a BR tightly interacts with sTM+HR1 segments, which sterically inhibits the large structural transition of the sTM+HR1 upwards. (**C**) Similar to the panel (B), but the sTM+HR1 fragment is removed. The dislocation of the sTM+HR1 fragment leaves a large empty space.

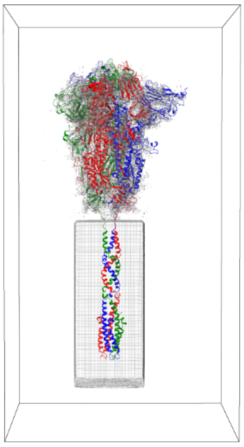

Figure S11. Cryo-EM density used for the constraint in the PRE structure modeling. The density map used as a constraint in Rosetta hybridize protocol to construct a full-length PRE structure. The density for the stalk region was removed from the Cryo-EM map (EMD-22292).

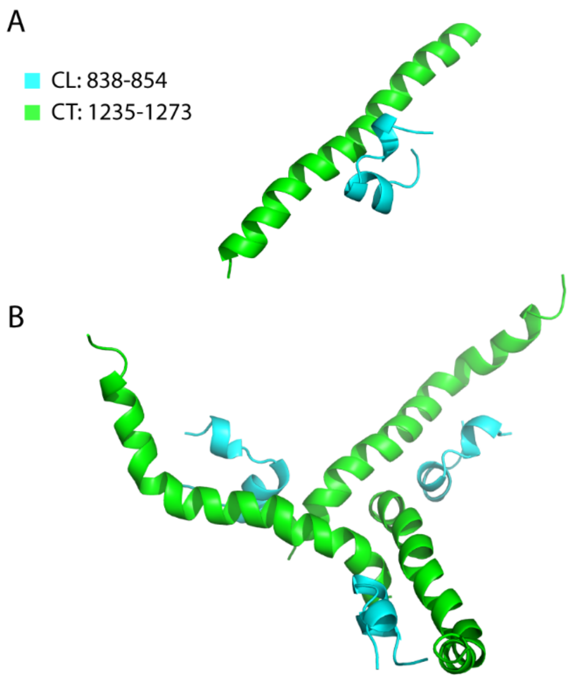

Figure S12. Optimized POST state structure in the cytoplasmic region. (**A**) Monomeric optimized structure of the cytoplasmic loop-cytoplasmic tail (CL-CT) segments complex. Cytoplasmic loop (CL) and cytoplasmic tail (CT) are shown in green and cyan, respectively. (**B**) Trimeric optimized structure. A C3 symmetric constraint was applied. The cytoplasmic complex is thought to work as the linchpin at the distal end of the uninterpreted density, restricting the possible conformation of the rest of the uninterpreted segments.

Table S1. Abbreviations used in this study.

Abbreviation	meaning
ACE2	Angiotensin-converting enzyme 2
BR	β-rich region
BCoV	Bovine coronavirus
CEACAM1	Carcinoembryonic antigen-related cell adhesion molecule 1
cH	Central helix
CT	Cytoplasmic tail
CTD	C-terminal domain
CL	Cytoplasmic loop
CoV	Coronavirus
COVID-19	Coronavirus disease 2019
Cryo-EM	Cryogenic electron microscopy
Cryo-ET	Cryogenic electron tomography
dFP	Downstream fusion peptide
DPP4	Dipeptidyl peptidase 4
eTM	Extended transmembrane
ER	Endoplasmic reticulum
FP	Fusion peptide
FPE model	Fusion peptide embedded model
HIV-1	Human Immunodeficiency Virus Type 1
HR1	Heptad repeat1
HR2	Heptad repeat2
INT1	Internal sub domain1
INT2	Internal sub domain2
L	Linker
LIC	Long inter core
LOC	Long outer core
LNP	Lipid nanoparticle
MAP Kinase	Mitogen-activated protein kinase
MERS-CoV	Middle East respiratory syndrome Coronavirus
MHV	Mouse hepatitis coronavirus
mRNA	Messenger ribonucleic acid
NMR	Nuclear magnetic resonance
NRP1	Neuropilin 1
NTD	N-terminal domain
ORF	Open Reading Frame
pTM	Pre-transmembrane
RAS	Renin-Angiotensin System
RBD	Receptor binding domain
RSV	Respiratory syncytial virus
SARS-CoV-1/2	Severe Acute Respiratory Syndrome Coronavirus 1/2
SE model	Stem embedded model
sTM	Sub-transmembrane
TM	Transmembrane
TMPRSS2	Transmembrane protease serine 2
uFP	Upstream fusion peptide
uH	Upstream helix
UPR	Unfolded protein response

Table S2. List of segments with previously reported functionality (fusogenic activity) in the literature. The list of literature and fusogenic segments that are validated by experiments is shown. The segments are mostly chosen from SARS-CoV-1 spike, and corresponding residue ids in SARS-CoV-2 spike are listed. We could not identify exact residue ids in a few cases. In summary, the fusogenic regions are 258-273, 633-650, 682-685, 788-906, 915-931, 1095-1110, 1203-1273. Based on the comprehensive idea of functional regions, some of the segments are reorganized in this study (Fig. 1A). The characteristics of segments are also considered to construct the computational structural models in this work.

Position SARS-CoV-2	Position SARS-CoV-1	Name in this study	Name in the Ref.	Ref.	Memo
788-806	770–778	uFP	SARS$_{\mathrm{WW-I}}$	[7]	FP
882-904	864–886	sTM	SARS$_{\mathrm{WW-II}}$	[7]	weak
816-833	798 - 815	dFP	S2 fusion peptide	[8], [9]	L803, L804, and F805
891-906	873-888	sTM	SARS$_{\mathrm{IFP}}$	[10]	Interact with phospholipids
Upstream of HR1	Upstream of HR1	sTM	Upstream of HR1	[11]	Infection inhibition
ca. 258-273	ca. 245-260	NTD	34	[12]	Obtained from Fig2b
ca. 633-650	ca. 619-636	CTD	85	[12]	Obtained from Fig2b
846-906	828-888	CL, sTM	R1 (113,119)	[12]	
876-904	858-886	sTM	FP in R1	[12]	
915-931	897-913	N-term of HR1	boundary in R1 (123)	[12]	
1095-1110	1077-1092	BR	R2 (147)	[12]	
1203-1228	1185-1210	pTM	R3 (162,163)	[12]	
788-806	770-788	dFP	SARS$_{\mathrm{FP}}$	[13]	
788-806	770-788	dFP	FP	[14], [9]	
891-906	873-888	sTM	IFP	[14], [9]	
1203-1220	1185-1202	pTM	PTM	[14], [9]	
873-898	855-880	sTM	pFP	[9], [15]	
776-839	758-821	uFP, dFP	LFP	[9]	
1205-1218	1187-1200	pTM	JMD	[16]	
1208-1222	1190–1204	pTM	Trp-rich region	[17]	
816-836	798-818	dFP	FP1	[18]	
834-853	816-835	CL	FP2	[18]	
835-855	817-837	CL	Cysteine-flanked region	[19]	Antigenic determinant
1235-1273	1217-1255	CT	endodomain	[20]	
1207-1256	1189-1238	pTM, TM	TMD	[21]	
1224-1273	1206-1255	TM, CT	CT	[22]	
682-685	NA	CendR	CendR	[23]	

Table S3. List of the publicly available spike protein computational models. To date, there are three downloadable structures of full-length spike protein for SARS-CoV-2. All of them are in the PRE state and computationally modeled incorporating Cryo-EM structures. There are more computational studies about the PRE state spikes reported in the literature.

State	URL	Tools	Ref.	Published
PRE	http://www.charmm-gui.org/docs/archive/covid19	GalaxyTBM, FALC, ISOLDE	[24]	2020-06-19
PRE	https://zhanglab.ccmb.med.umich.edu/COVID-19/	C-I-TASSER	NA	
PRE	https://doi.org/10.1021/acscentsci.0c01056 https://amarolab.ucsd.edu/covid19.php	Modeller, i-TASSER	[25]	2020-09-23

Table S4. Examples of rotational motion of an S1 trimer. The rotational motions along the 3-fold axis were reported in the literature. They are associated with RDB Up/Down and different degrees. Direction of the S1 rotation is analyzed against the 3-fold rotation axis from an S1 trimer side. * the rotation is mainly attributed dihedral angle φ_2 (see the ref [26] for the definition).

Virus	State of Structure1	EMD ID / PDB ID 1	State of Structure2	EMD ID / PDB ID 2	S1s rotation (struc1 to struc2)	Ref.
SARS-CoV-2	Down	EMD-22292 / 6XR8	Up	EMD-21457 / 6VYB	Clockwise	[27]
SARS-CoV-2	Down	EMDB-11207 / 6ZGI	Intermediate	EMDB-11206 / 6ZGH	Clockwise	[28]
SARS-CoV-2	Tightly Down	EMD-30701 / 7DF3	Down	EMD-21452 / 6VXX	Clockwise	[29]
SARS-CoV-2	Tightly Down	EMD-30701 / 7DF3	Up	EMD-30701 / 7DK3	Clockwise	[29]
SARS-CoV-2	Down	Ensemble of Down	Up	Ensemble of Up	Clockwise *	[26]
SARS-CoV-1	No ligand (Down)	EMD-1423	ACE2 bind (Up)	EMD-1425	Clockwise	[30]

Table S5. List of deposited EMDB density maps and PDB structures. There are more than 300 Cryo-EM PRE state structures. For simplicity and visuality, only relevant coronavirus spikes in the POST state are listed.

Virus	State	EMD ID / PDB ID	Resolution	Ref.	Released
MHV	Post-fusion	EMD-7040 / 6B3O	4.1	[31]	2017-10
SARS-CoV	Post-fusion	EMD-9597	30.5	[4]	2018-08
SARS-CoV-2	Post-fusion	EMD-11627	10.7	[32]	2020-10
SARS-CoV-2	Post-fusion	EMD-22293 / 6XRA	3.0	[27]	2020-07
SARS-CoV	Post-fusion	EMD-30072 / 6M3W	3.9	[33]	2020-06
SARS-CoV-2	Post-fusion	EMD-30428	15.3	[34]	2020-09

Table S6. Summary of the population in extended (INT/POST) state on the SARS-CoV-2 virion.

Ref.	Population of extended (INT/POST) state	Cell culture	Inactivation	Memo
[35]	< 0.1 %	Vero E6	paraformaldehyde	
[34]	18%	Vero	paraformaldehyde	
[36]	almost 100%	A549 + ACE2	paraformaldehyde	ACE2 dependency is possible
	Mixture	Vero E6		
	Almost 0%	Calu3		
[37]	3%	Vero E6, Calu3	formaldehyde	Purification method can alter the ratio of PRE conformation
[32]	74.4%		β-propiolactone	S1 is partly present (Fig. S2c)

Table S7. List of putative host factors including receptors and attachment factors. The host factors affecting infectivity are listed. Note they are mostly considered to serve as a receptor and their receptor binding sites are located in S1. (For review [38], [39]). * computational study. For comprehensive factors, see Table S2 in [40].

Virus	Receptor/effecter	Binding site	Ref.
SARS-CoV-2	ACE2	S1 RDB	[41], [42]
SARS-CoV-2	NRP1	S1 CendR	[43], [44]
SARS-CoV-2	heparin sulfate	S1 RDB*	[45], [46]
SARS-CoV-2	Sugar?, protein?	S1 NTD (GTNGTKR motif)	[47]
SARS-CoV-2	Sialic acid	S1 NTD	[48]
HCoV-OC43	9-O-acetylated sialic acid	A domain	[49]
SARS-CoV-2	BSG, CD147, BASIGIN	S1 RBD	[50]
SARS-CoV-2	GRP78	S1 RBD*	[51]*
MARS-CoV, bCoV-HKU9	GRP78	Spike RBD	[52]
SARS-CoV-2	lipid	S1 RBD	[53]
SARS-CoV-2	Ganglioside	S1 NTD	[54]
SARS-CoV-2	cholesterol		[55], [56]
SARS-CoV-2	Ezlin	CT	[20]
SARS-CoV-1	Ca^{2+}	S2 (fusion peptide)	[18]
SARS-CoV-2	IFITM, IFITM1, IFITM2, IFITM3		[57], [58]

Table S8. List of enzymes reported with the cleavage sites.

Virus	S1/S2	S2′	Ref.
SARS-CoV-1	Trypsin, Cathepsin L		[59]
SARS-CoV-2	Furin, PC1, Trypsin, Cathepsin L, Cathepsin B		[59]
SARS-CoV-2	Furin	TMPRSS2	[60]
SARS-CoV-1	Cathepsin L, trypsin, Thermolysin, TMPRSS11d (HAT), Factor Xa	Elastase	[61], [62]
SARS-CoV-2	Furin		[63],[64]
SARS-CoV-1, MARS-CoV	Not Furin		[64]
SARS-CoV-1	Trypsin	TMPRSS2	[65]
SARS-CoV-2	Cathepsin L<br>Not Cathepsin B		[66]
SARS-CoV-1	Furin, Cathepsin L		[67]
SARS-CoV-1, SARS-CoV-2		TMPRSS11D, TMPRSS13	[68]

Table S9. Summary of experimental conditions that are successful in obtaining atomic SARS-CoV-1/2 spike structure in POST state in literature. It is noteworthy that the situations of the spike (e.g., S1 shedding) are slightly different depending on the experimental conditions. The cleavage of S1/S2 and S2', and up/down of RBD appears to depend on the enzyme and soluble ACE2 concentrations, respectively. Also, ectodomain spike may not require S2' cleavage for the POST transition.

Ref.	Virus	Condition	Memo
[33]	SARS-CoV-1	With soluble ACE2 (1:20), trypsin, spike ectodomain, low pH 5.5	S1 is absent (emd_30072)
[27]	SARS-CoV-2	With soluble ACE2 (1:10) and NP-40 (detergent)	S1 is absent (Fig.4C)<br>S2' is cleaved (Fig.S8)
[4]	SARS-CoV-1	With soluble ACE2 (1:3), trypsin, spike ectodomain, low pH 5.6	S1 is present (Fig.3D)

Table S10. Comparison of viral fusion mechanism between our model and the conventional model mediated by class I fusion protein. The limited items are listed to compare.

	**Proposed mechanism in this work**	**Traditional descriptions mediated by class I fusion protein**
Structural states	PRE, INT, POST	Pre-fusion, Pre-hairpin, Fold-back, Post-fusion
Unresolved structures	INT	Pre-hairpin<br>Hairpin<br>Fold-back
Viral transmembrane region of POST/post-fusion state on the virion	Stem (SE model)	TM and dFP (FPE model)
Timing of S1 dissociation	Induced by S2′ cleavage (after the structural transition)	Before the structural transition
Role of S1	Receptor binding and determinant of the membrane fusion	Receptor binding
Role of S1/S2-S2′	Stabilizing the stem in the viral membrane. Making a smooth curvature of fused membrane surface	Not discussed
Position of CT during the transition	Move from the viral to host membrane	Always in the viral membrane
The number of spike protein required for membrane fusion	Any	Multiple, but not large number
Cooperativity for the structural transition among spikes	No restriction or concerted S1 trimers release	Concerted structural transition
Distribution for membrane fusion	No restriction	Aligned around the pore
Location of the spike after the membrane fusion	S1, S1/S2-S2′, S2′ subunits, mediating the fusion, pass through the host membrane	S1 sheds. S2′ ectodomain is in the extracellular region or extrasellar vesicle, membrane-bound

Table S11. List of software used in this study.

Ref.	Name of the software	Protocol	Purpose
[69]	Rosetta	Symmetric Docking	Stalk modeling for the PRE state
[70]	Rosetta	Hybridize	Combine smaller fragments
[71]	NAMD	Molecular Dynamics	Flexible fitting
[72]	Rosetta	Docking	To model the CL-CT complex
[73]	Rosetta+PyRosetta	TrRosetta	To model small fragments
[74]	Rosetta	*Ab-initio* relax	To model small fragments

Table S12. Resolution and sampling statistics for the original domains. List of the domains used/modeled to further hybridize the full-length computational models (PRE, POST structures). The Cryo-EM maps (EMD-22292, EMD-9597) are used for the overall shape constraints to apply in the PRE, POST structural modeling, respectively. The partial disordered region for Cryo-EM residue ids is not described.

State	Domain	Residue ids	Source/Method	Source ID	Resolution/Sampling
PRE	Ectodomain	14-1162	Cryo-EM	PDB: 6XR8	resolution 2.9 Å
PRE	Stalk	1114-1273	Rosetta dock + hybridization	NA	over 114,000 docking structures<br>over 305,000 fragments sampled in total
POST	S1	1-685	Cryo-EM	PDB: 6VSB	resolution: 3.5 Å
POST	Main	706-771<br>919-1196	Cryo-EM	PDB: 6M3W	resolution: 3.9 Å
POST	Main	703-770,<br>912-1197	Cryo-EM	PDB: 6XRA	resolution: 3.0 Å
POST	N-term of S1/S2-S2′	667-705	TrRosetta	NA	top 5 structures are provided online
POST	C-term of S1/S2-S2′	797-815	Rosetta *ab-initio*	NA	over 32,000 structures sampled
POST	CT-CL monomer	838-845,<br>1237-1273	Rosetta dock	NA	over 24,000 structures sampled
POST	CT-CL trimer	838-845,<br>1237-1273	Rosetta dock	NA	over 32,000 structures sampled

References

1. Tsirigos, K.; Peters, C.; Shu, N.; Käll, L.; Elofsson, A. The TOPCONS web server for consensus prediction of membrane protein topology and signal peptides. *Nucleic Acids Res.* **2015**, *43*, W401–W407, doi:10.1093/nar/gkv485.
2. Nakamura, T.; Yamada, K.D.; Tomii, K.; Katoh, K. Parallelization of MAFFT for large-scale multiple sequence alignments. *Bioinform.* **2018**, *34*, 2490–2492, doi:10.1093/bioinformatics/bty121.
3. Gouet, P. ESPript/ENDscript: extracting and rendering sequence and 3D information from atomic structures of proteins. *Nucleic Acids Res.* **2003**, *31*, 3320–3323, doi:10.1093/nar/gkg556.
4. Song, W.; Gui, M.; Wang, X.; Xiang, Y. Cryo-EM structure of the SARS coronavirus spike glycoprotein in complex with its host cell receptor ACE2. *PLoS Pathog.* **2018**, *14*, e1007236, doi:10.1371/journal.ppat.1007236.
5. Jurrus, E.; Engel, D.; Star, K.; Monson, K.; Brandi, J.; Felberg, L.E.; Brookes, D.H.; Wilson, L.; Chen, J.; Liles, K.; et al. Improvements to the APBS biomolecular solvation software suite. *Protein Sci.* **2018**, *27*, 112–128, doi:10.1002/pro.3280.
6. Dolinsky, T.J.; Czodrowski, P.; Li, H.; Nielsen, J.E.; Jensen, J.H.; Klebe, G.; Baker, N.A. PDB2PQR: expanding and upgrading automated preparation of biomolecular structures for molecular simulations. *Nucleic Acids Res.* **2007**, *35*, W522–W525, doi:10.1093/nar/gkm276.
7. Sainz, B.; Rausch, J.M.; Gallaher, W.R.; Garry, R.; Wimley, W.C. Identification and Characterization of the Putative Fusion Peptide of the Severe Acute Respiratory Syndrome-Associated Coronavirus Spike Protein. *J. Virol.* **2005**, *79*, 7195–7206, doi:10.1128/jvi.79.11.7195-7206.2005.
8. Madu, I.G.; Roth, S.L.; Belouzard, S.; Whittaker, G.R. Characterization of a Highly Conserved Domain within the Severe Acute Respiratory Syndrome Coronavirus Spike Protein S2 Domain with Characteristics of a Viral Fusion Peptide. *J. Virol.* **2009**, *83*, 7411–7421, doi:10.1128/jvi.00079-09.
9. Mahajan, M.; Chatterjee, D.; Bhuvaneswari, K.; Pillay, S.; Bhattacharjya, S. NMR structure and localization of a large fragment of the SARS-CoV fusion protein: Implications in viral cell fusion. *Biochim. et Biophys. Acta (BBA) - Biomembr.* **2018**, *1860*, 407–415, doi:10.1016/j.bbamem.2017.10.002.
10. Guillén, J.; Pérez-Berná, A.J.; Moreno, M.R.; Villalaín, J. A Second SARS-CoV S2 Glycoprotein Internal Membrane-Active Peptide. Biophysical Characterization and Membrane Interaction. *Biochem.* **2008**, *47*, 8214–8224, doi:10.1021/bi800814q.
11. Bosch, B.J.; Martina, B.E.E.; van der Zee, R.; Lepault, J.; Haijema, B.J.; Versluis, C.; Heck, A.; de Groot, R.; Osterhaus, A.; Rottier, P.J.M. Severe acute respiratory syndrome coronavirus (SARS-CoV) infection inhibition using spike protein heptad repeat-derived peptides. *Proc. Natl. Acad. Sci.* **2004**, *101*, 8455–8460, doi:10.1073/pnas.0400576101.
12. Guille´n, J.; Pe´rez-Berna, A.; Moreno, M.R.; Villalaı´n, J. Identification of the Membrane-Active Regions of the Severe Acute Respiratory Syndrome Coronavirus Spike Membrane Glycoprotein Using a 16/18-Mer Peptide Scan: Implications for the Viral Fusion Mechanism. *J. Virol.* **2005**, *79*, 1743–1752. 10.1128/JVI.79.3.1743.
13. Guillén, J.; de Almeida, R.F.M.; Prieto, M.; Villalaín, J. Structural and Dynamic Characterization of the Interaction of the Putative Fusion Peptide of the S2 SARS-CoV Virus Protein with Lipid Membranes. *J. Phys. Chem. B* **2008**, *112*, 6997–7007, doi:10.1021/jp7118229.
14. Mahajan, M.; Bhattacharjya, S. NMR structures and localization of the potential fusion peptides and the pre-transmembrane region of SARS-CoV: Implications in membrane fusion. *Biochim. et Biophys. Acta (BBA) - Biomembr.* **2015**, *1848*, 721–730, doi:10.1016/j.bbamem.2014.11.025.
15. Ou, X.; Zheng, W.; Shan, Y.; Mu, Z.; Dominguez, S.R.; Holmes, K.V.; Qian, Z. Identification of the Fusion Peptide-Containing Region in Betacoronavirus Spike Glycoproteins. *J. Virol.* **2016**, *90*, 5586–5600, doi:10.1128/jvi.00015-16.
16. Howard, M.W.; Travanty, E.A.; Jeffers, S.A.; Smith, M.K.; Wennier, S.T.; Thackray, L.B.; Holmes, K.V. Aromatic Amino Acids in the Juxtamembrane Domain of Severe Acute Respiratory Syndrome Coronavirus Spike Glycoprotein Are Important for Receptor-Dependent Virus Entry and Cell-Cell Fusion. *J. Virol.* **2008**, *82*, 2883–2894, doi:10.1128/jvi.01805-07.
17. Lu, Y.; Neo, T.L.; Liu, D.X.; Tam, J.P. Importance of SARS-CoV spike protein Trp-rich region in viral infectivity. *Biochem. Biophys. Res. Commun.* **2008**, *371*, 356–360, doi:10.1016/j.bbrc.2008.04.044.
18. Lai, A.L.; Millet, J.K.; Daniel, S.; Freed, J.H.; Whittaker, G.R. The SARS-CoV Fusion Peptide Forms an Extended Bipartite Fusion Platform that Perturbs Membrane Order in a Calcium- Dependent Manner. *J Mol Biol* **2017**, *429*, 3875–3892.
19. Madu, I.G.; Belouzard, S.; Whittaker, G.R. SARS-coronavirus spike S2 domain flanked by cysteine residues C822 and C833 is important for activation of membrane fusion. *Virol. J.* **2009**, *393*, 265–271, doi:10.1016/j.virol.2009.07.038.
20. Millet, J.K.; Kien, F.; Cheung, C.-Y.; Siu, Y.-L.; Chan, W.-L.; Li, H.; Leung, H.-L.; Jaume, M.; Bruzzone, R.; Peiris, J.S.M.; et al. Ezrin Interacts with the SARS Coronavirus Spike Protein and Restrains Infection at the Entry Stage. *PLoS ONE* **2012**, *7*, e49566, doi:10.1371/journal.pone.0049566.
21. Corver, J.; Broer, R.; Van Kasteren, P.; Spaan, W.J.M. Mutagenesis of the transmembrane domain of the SARS coronavirus spike glycoprotein: refinement of the requirements for SARS coronavirus cell entry. *Virol. J.* **2009**, *6*, 230, doi:10.1186/1743-422x-6-230.
22. Petit, C.M.; Chouljenko, V.N.; Iyer, A.; Colgrove, R.; Farzan, M.; Knipe, D.M.; Kousoulas, K. Palmitoylation of the cysteine-rich endodomain of the SARS–coronavirus spike glycoprotein is important for spike-mediated cell fusion. *Virol. J.* **2007**, *360*, 264–274, doi:10.1016/j.virol.2006.10.034.
23. Teesalu, T.; Sugahara, K.N.; Kotamraju, V.R.; Ruoslahti, E. C-end rule peptides mediate neuropilin-1-dependent cell, vascular, and tissue penetration. *Proc. Natl. Acad. Sci.* **2009**, *106*, 16157–16162, doi:10.1073/pnas.0908201106.

24. Woo, H.; Park, S.-J.; Choi, Y.K.; Park, T.; Tanveer, M.; Cao, Y.; Kern, N.R.; Lee, J.; Yeom, M.S.; Croll, T.I.; et al. Developing a Fully Glycosylated Full-Length SARS-CoV-2 Spike Protein Model in a Viral Membrane. *J. Phys. Chem. B* **2020**, *124*, 7128–7137, doi:10.1021/acs.jpcb.0c04553.
25. Casalino, L.; Gaieb, Z.; Goldsmith, J.A.; Hjorth, C.K.; Dommer, A.C.; Harbison, A.M.; Fogarty, C.A.; Barros, E.P.; Taylor, B.C.; McLellan, J.S.; et al. Beyond Shielding: The Roles of Glycans in the SARS-CoV-2 Spike Protein. *ACS Central Sci.* **2020**, *6*, 1722–1734, doi:10.1021/acscentsci.0c01056.
26. Henderson, R.; Edwards, R.J.; Mansouri, K.; Janowska, K.; Stalls, V.; Gobeil, S.M.C.; Kopp, M.; Li, D.; Parks, R.; Hsu, A.L.; et al. Controlling the SARS-CoV-2 spike glycoprotein conformation. *Nat. Struct. Mol. Biol.* **2020**, *27*, 925–933, doi:10.1038/s41594-020-0479-4.
27. Cai, Y.; Zhang, J.; Xiao, T.; Peng, H.; Sterling, S.M.; Jr, R.M.W.; Rawson, S.; Rits-Volloch, S.; Chen, B. Distinct conformational states of SARS-CoV-2 spike protein. *Science* **2020**, *369*, 1586–1592, doi:10.1126/science.abd4251.
28. Wrobel, A.G.; Benton, D.J.; Xu, P.; Roustan, C.; Martin, S.R.; Rosenthal, P.B.; Skehel, J.J.; Gamblin, S.J. SARS-CoV-2 and bat RaTG13 spike glycoprotein structures inform on virus evolution and furin-cleavage effects. *Nat. Struct. Mol. Biol.* **2020**, *27*, 763–767, doi:10.1038/s41594-020-0468-7.
29. Xu, C.; Wang, Y.; Liu, C.; Zhang, C.; Han, W.; Hong, X.; Wang, Y.; Hong, Q.; Wang, S.; Zhao, Q.; et al. Conformational dynamics of SARS-CoV-2 trimeric spike glycoprotein in complex with receptor ACE2 revealed by cryo-EM. *Sci. Adv.* **2020**, *5575*, 1–20. 10.1101/2020.06.30.177097.
30. Beniac, D.R.; Devarennes, S.L.; Andonov, A.; He, R.; Booth, T.F. Conformational Reorganization of the SARS Coronavirus Spike Following Receptor Binding: Implications for Membrane Fusion. *PLoS ONE* **2007**, *2*, e1082, doi:10.1371/journal.pone.0001082.
31. Walls, A.; Tortorici, M.A.; Snijder, J.; Xiong, X.; Bosch, B.-J.; Rey, F.A.; Veesler, D. Tectonic conformational changes of a coronavirus spike glycoprotein promote membrane fusion. *Proc. Natl. Acad. Sci.* **2017**, *114*, 11157–11162, doi:10.1073/pnas.1708727114.
32. Liu, C.; Mendonça, L.; Yang, Y.; Gao, Y.; Shen, C.; Liu, J.; Ni, T.; Ju, B.; Liu, C.; Tang, X.; et al. The Architecture of Inactivated SARS-CoV-2 with Postfusion Spikes Revealed by CryoEM and CryoET. *Structure* **2020**, *28*, 1–7. 10.2139/ssrn.3659985.
33. Fan, X.; Cao, D.; Kong, L.; Zhang, X. Cryo-EM analysis of the post-fusion structure of the SARS-CoV spike glycoprotein. *Nat. Commun.* **2020**, *11*, 1–10, doi:10.1038/s41467-020-17371-6.
34. Yao, H.; Song, Y.; Chen, Y.; Wu, N.; Xu, J.; Sun, C.; Zhang, J.; Weng, T.; Zhang, Z.; Wu, Z.; et al. Molecular Architecture of the SARS-CoV-2 Virus. *Cell* **2020**, *183*, 730–738.e13, doi:10.1016/j.cell.2020.09.018.
35. Turoňová, B.; Sikora, M.; Schürmann, C.; Hagen, W.J.H.; Welsch, S.; Blanc, F.E.C.; Von Bülow, S.; Gecht, M.; Bagola, K.; Hörner, C.; et al. In situ structural analysis of SARS-CoV-2 spike reveals flexibility mediated by three hinges. *Science* **2020**, *370*, eabd5223–208, doi:10.1126/science.abd5223.
36. Klein, S.; Cortese, M.; Winter, S.L.; Wachsmuth-Melm, M.; Neufeldt, C.J.; Cerikan, B.; Stanifer, M.L.; Boulant, S.; Bartenschlager, R.; Chlanda, P. SARS-CoV-2 structure and replication characterized by in situ cryo-electron tomography. *Nat. Commun.* **2020**, *11*, 5885, doi:10.1038/s41467-020-19619-7.
37. Ke, Z.; Oton, J.; Qu, K.; Cortese, M.; Zila, V.; McKeane, L.; Nakane, T.; Zivanov, J.; Neufeldt, C.J.; Cerikan, B.; et al. Structures and distributions of SARS-CoV-2 spike proteins on intact virions. *Nat. Cell Biol.* **2020**, *588*, 498–502, doi:10.1038/s41586-020-2665-2.
38. Cuervo, N.Z.; Grandvaux, N. ACE2: Evidence of role as entry receptor for SARS-CoV-2 and implications in comorbidities. *eLife* **2020**, *9*, 1-, doi:10.7554/elife.61390.
39. Kočar, E.; Režen, T.; Rozman, D. Cholesterol, lipoproteins, and COVID-19: Basic concepts and clinical applications. *Biochim. et Biophys. Acta (BBA) - Mol. Cell Biol. Lipids* **2021**, *1866*, 158849, doi:10.1016/j.bbalip.2020.158849.
40. Singh, M.; Bansal, V.; Feschotte, C. A Single-Cell RNA Expression Map of Human Coronavirus Entry Factors. *Cell Rep.* **2020**, *32*, 108175, doi:10.1016/j.celrep.2020.108175.
41. Lan, J.; Ge, J.; Yu, J.; Shan, S.; Zhou, H.; Fan, S.; Zhang, Q.; Shi, X.; Wang, Q.; Zhang, L.; et al. Structure of the SARS-CoV-2 spike receptor-binding domain bound to the ACE2 receptor. *Nat. Cell Biol.* **2020**, *581*, 215–220, doi:10.1038/s41586-020-2180-5.
42. Hoffmann, M.; Kleine-Weber, H.; Schroeder, S.; Krüger, N.; Herrler, T.; Erichsen, S.; Schiergens, T.S.; Herrler, G.; Wu, N.-H.; Nitsche, A.; et al. SARS-CoV-2 Cell Entry Depends on ACE2 and TMPRSS2 and Is Blocked by a Clinically Proven Protease Inhibitor. *Cell* **2020**, *181*, 271–280.e8, doi:10.1016/j.cell.2020.02.052.
43. Cantuti-Castelvetri, L.; Ojha, R.; Pedro, L.D.; Djannatian, M.; Franz, J.; Kuivanen, S.; Van Der Meer, F.; Kallio, K.; Kaya, T.; Anastasina, M.; et al. Neuropilin-1 facilitates SARS-CoV-2 cell entry and infectivity. *Science* **2020**, *370*, 856–860, doi:10.1126/science.abd2985.
44. Daly, J.; Simonetti, B.; Antón-Plágaro, C.; Kavanagh Williamson, M.; Shoemark, D.; Simón-Gracia, L.; Klein, K.; Bauer, M.; Hollandi, R.; Greber, U.; et al. Neuropilin-1 is a host factor for SARS-CoV-2 infection. *Science* **2020**, *3072*, 1–8. 10.1101/2020.06.05.134114.
45. Clausen, T.M.; Sandoval, D.R.; Spliid, C.B.; Pihl, J.; Perrett, H.R.; Painter, C.D.; Narayanan, A.; Majowicz, S.A.; Kwong, E.M.; McVicar, R.N.; et al. SARS-CoV-2 Infection Depends on Cellular Heparan Sulfate and ACE2. *Cell* **2020**, *183*, 1043–1057.e15, doi:10.1016/j.cell.2020.09.033.
46. Kim, S.Y.; Jin, W.; Sood, A.; Montgomery, D.W.; Grant, O.C.; Fuster, M.M.; Fu, L.; Dordick, J.S.; Woods, R.J.; Zhang, F.; et al. Characterization of heparin and severe acute respiratory syndrome-related coronavirus 2 (SARS-CoV-2) spike glycoprotein binding interactions. *Antivir. Res.* **2020**, *181*, 104873, doi:10.1016/j.antiviral.2020.104873.

47. Behloul, N.; Baha, S.; Shi, R.; Meng, J. Role of the GTNGTKR motif in the N-terminal receptor-binding domain of the SARS-CoV-2 spike protein. *Virus Res.* **2020**, *286*, 198058, doi:10.1016/j.virusres.2020.198058.
48. Engin, A.B.; Engin, E.D.; Engin, A. Dual function of sialic acid in gastrointestinal SARS-CoV-2 infection. *Environ. Toxicol. Pharmacol.* **2020**, *79*, 103436, doi:10.1016/j.etap.2020.103436.
49. Tortorici, M.A.; Walls, A.C.; Lang, Y.; Wang, C.; Li, Z.; Koerhuis, D.; Boons, G.-J.; Bosch, B.-J.; Rey, F.A.; De Groot, R.J.; et al. Structural basis for human coronavirus attachment to sialic acid receptors. *Nat. Struct. Mol. Biol.* **2019**, *26*, 481–489, doi:10.1038/s41594-019-0233-y.
50. Wang, K.; Chen, W.; Zhang, Z.; Deng, Y.; Lian, J.-Q.; Du, P.; Wei, D.; Zhang, Y.; Sun, X.-X.; Gong, L.; et al. CD147-spike protein is a novel route for SARS-CoV-2 infection to host cells. *Signal Transduct. Target. Ther.* **2020**, *5*, 1–10, doi:10.1038/s41392-020-00426-x.
51. Ibrahim, I.M.; Abdelmalek, D.H.; Elshahat, M.E.; Elfiky, A.A. COVID-19 spike-host cell receptor GRP78 binding site prediction. *J. Infect.* **2020**, *80*, 554–562, doi:10.1016/j.jinf.2020.02.026.
52. Chu, H.; Chan, C.-M.; Zhang, X.; Wang, Y.; Yuan, S.; Zhou, J.; Au-Yeung, R.K.-H.; Sze, K.-H.; Yang, D.; Shuai, H.; et al. Middle East respiratory syndrome coronavirus and bat coronavirus HKU9 both can utilize GRP78 for attachment onto host cells. *J. Biol. Chem.* **2018**, *293*, 11709–11726, doi:10.1074/jbc.ra118.001897.
53. Toelzer, C.; Gupta, K.; Yadav, S.K.N.; Borucu, U.; Davidson, A.D.; Williamson, M.K.; Shoemark, D.K.; Garzoni, F.; Staufer, O.; Milligan, R.; et al. Free fatty acid binding pocket in the locked structure of SARS-CoV-2 spike protein. *Science* **2020**, *370*, 725–730, doi:10.1126/science.abd3255.
54. Fantini, J.; Chahinian, H.; Yahi, N. Leveraging coronavirus binding to gangliosides for innovative vaccine and therapeutic strategies against COVID-19. *Biochem. Biophys. Res. Commun.* **2021**, *538*, 132–136, doi:10.1016/j.bbrc.2020.10.015.
55. Wei, C.; Wan, L.; Yan, Q.; Wang, X.; Zhang, J.; Yang, X.; Zhang, Y.; Fan, C.; Li, D.; Deng, Y.; et al. HDL-scavenger receptor B type 1 facilitates SARS-CoV-2 entry. *Nat. Metab.* **2020**, *2*, 1391–1400, doi:10.1038/s42255-020-00324-0.
56. Sanders, D.W.; Jumper, C.C.; Ackerman, P.J.; Bracha, D.; Donlic, A.; Kim, H.; Kenney, D.; Castello-Serrano, I.; Suzuki, S.; Tamura, T.; et al. SARS-CoV-2 Requires Cholesterol for Viral Entry and Pathological Syncytia Formation. bioRxiv **2020**, 2020.12.14.422737.
57. Shi, G.; Kenney, A.D.; Kudryashova, E.; Zani, A.; Zhang, L.; Lai, K.K.; Hall-Stoodley, L.; Robinson, R.T.; Kudryashov, D.S.; Compton, A.A.; et al. Opposing activities of IFITM proteins in SARS-CoV-2 infection. *EMBO J.* **2021**, *40*, e106501, doi:10.15252/embj.2020106501.
58. Bozzo, C.P.; Nchioua, R.; Volcic, M.; Krüger, J.; Heller, S.; Stürzel, C.M.; Kmiec, D.; Conzelmann, C.; Müller, J.; Zech, F.; et al. IFITM proteins promote SARS-CoV-2 infection and are targets for virus inhibition. bioRxiv **2020**, 2020.08.18.255935. 10.1101/2020.08.18.255935.
59. Jaimes, J.A.; Millet, J.K.; Whittaker, G.R. Proteolytic Cleavage of the SARS-CoV-2 Spike Protein and the Role of the Novel S1/S2 Site. *iScience* **2020**, *23*, 101212, doi:10.1016/j.isci.2020.101212.
60. Bestle, D.; Heindl, M.R.; Limburg, H.; Van, T.V.L.; Pilgram, O.; Moulton, H.; A Stein, D.; Hardes, K.; Eickmann, M.; Dolnik, O.; et al. TMPRSS2 and furin are both essential for proteolytic activation of SARS-CoV-2 in human airway cells. *Life Sci. Alliance* **2020**, *3*, e202000786, doi:10.26508/lsa.202000786.
61. Millet, J.K.; Whittaker, G.R. Host cell proteases: Critical determinants of coronavirus tropism and pathogenesis. *Virus Res.* **2015**, *202*, 120–134, doi:10.1016/j.virusres.2014.11.021.
62. Belouzard, S.; Madu, I.; Whittaker, G.R. Elastase-mediated Activation of the Severe Acute Respiratory Syndrome Coronavirus Spike Protein at Discrete Sites within the S2 Domain. *J. Biol. Chem.* **2010**, *285*, 22758–22763, doi:10.1074/jbc.m110.103275.
63. Hoffmann, M.; Kleine-Weber, H.; Pöhlmann, S. A Multibasic Cleavage Site in the Spike Protein of SARS-CoV-2 Is Essential for Infection of Human Lung Cells. *Mol. Cell* **2020**, *78*, 779–784.e5, doi:10.1016/j.molcel.2020.04.022.
64. Örd, M.; Faustova, I.; Loog, M. The sequence at Spike S1/S2 site enables cleavage by furin and phospho-regulation in SARS-CoV2 but not in SARS-CoV1 or MERS-CoV. *Sci. Rep.* **2020**, *10*, 1–10, doi:10.1038/s41598-020-74101-0.
65. Reinke, L.M.; Spiegel, M.; Plegge, T.; Hartleib, A.; Nehlmeier, I.; Gierer, S.; Hoffmann, M.; Hofmann-Winkler, H.; Winkler, M.; Pöhlmann, S. Different residues in the SARS-CoV spike protein determine cleavage and activation by the host cell protease TMPRSS2. *PLoS ONE* **2017**, *12*, e0179177, doi:10.1371/journal.pone.0179177.
66. Ou, X.; Liu, Y.; Lei, X.; Li, P.; Mi, D.; Ren, L.; Guo, L.; Guo, R.; Chen, T.; Hu, J.; et al. Characterization of spike glycoprotein of SARS-CoV-2 on virus entry and its immune cross-reactivity with SARS-CoV. *Nat. Commun.* **2020**, *11*, 1–12, doi:10.1038/s41467-020-15562-9.
67. Bosch, B.J.; Bartelink, W.; Rottier, P.J.M. Cathepsin L Functionally Cleaves the Severe Acute Respiratory Syndrome Coronavirus Class I Fusion Protein Upstream of Rather than Adjacent to the Fusion Peptide. *J. Virol.* **2008**, *82*, 8887–8890, doi:10.1128/jvi.00415-08.
68. Kishimoto, M.; Uemura, K.; Sanaki, T.; Sato, A.; Hall, W.; Kariwa, H.; Orba, Y.; Sawa, H.; Sasaki, M. TMPRSS11D and TMPRSS13 Activate the SARS-CoV-2 Spike Protein. *Viruses* **2021**, *13*, 384, doi:10.3390/v13030384.
69. André, I.; Bradley, P.; Wang, C.; Baker, D. Prediction of the structure of symmetrical protein assemblies. *Proc. Natl. Acad. Sci.* **2007**, *104*, 17656–17661, doi:10.1073/pnas.0702626104.
70. Song, Y.; DiMaio, F.; Wang, R.Y.-R.; Kim, D.; Miles, C.; Brunette, T.; Thompson, J.; Baker, D. High-Resolution Comparative Modeling with RosettaCM. *Struct.* **2013**, *21*, 1735–1742, doi:10.1016/j.str.2013.08.005.

71. Phillips, J.C.; Hardy, D.J.; Maia, J.D.C.; Stone, J.E.; Ribeiro, J.V.; Bernardi, R.C.; Buch, R.; Fiorin, G.; Hénin, J.; Jiang, W.; et al. Scalable molecular dynamics on CPU and GPU architectures with NAMD. *J. Chem. Phys.* **2020**, *153*, 044130, doi:10.1063/5.0014475.
72. Chaudhury, S.; Berrondo, M.; Weitzner, B.D.; Muthu, P.; Bergman, H.; Gray, J.J. Benchmarking and Analysis of Protein Docking Performance in Rosetta v3.2. *PLOS ONE* **2011**, *6*, e22477, doi:10.1371/journal.pone.0022477.
73. Yang, J.; Anishchenko, I.; Park, H.; Peng, Z.; Ovchinnikov, S.; Baker, D. Improved protein structure prediction using predicted interresidue orientations. *Proc. Natl. Acad. Sci.* **2020**, *117*, 1496–1503, doi:10.1073/pnas.1914677117.
74. Bradley, P.; Misura, K.M.S.; Baker, D. Toward High-Resolution de Novo Structure Prediction for Small Proteins. *Science* **2005**, *309*, 1868–1871, doi:10.1126/science.1113801.
